# Evaluation of the Gini Coefficient in Spatial Scan Statistics for Detecting Irregularly Shaped Clusters

**DOI:** 10.1371/journal.pone.0170736

**Published:** 2017-01-27

**Authors:** Jiyu Kim, Inkyung Jung

**Affiliations:** Department of Biostatistics and Medical Informatics, Yonsei University College of Medicine, Seoul, Korea; Stony Brook University, Graduate Program in Public Health, UNITED STATES

## Abstract

Spatial scan statistics with circular or elliptic scanning windows are commonly used for cluster detection in various applications, such as the identification of geographical disease clusters from epidemiological data. It has been pointed out that the method may have difficulty in correctly identifying non-compact, arbitrarily shaped clusters. In this paper, we evaluated the Gini coefficient for detecting irregularly shaped clusters through a simulation study. The Gini coefficient, the use of which in spatial scan statistics was recently proposed, is a criterion measure for optimizing the maximum reported cluster size. Our simulation study results showed that using the Gini coefficient works better than the original spatial scan statistic for identifying irregularly shaped clusters, by reporting an optimized and refined collection of clusters rather than a single larger cluster. We have provided a real data example that seems to support the simulation results. We think that using the Gini coefficient in spatial scan statistics can be helpful for the detection of irregularly shaped clusters.

## Introduction

Among various statistical methods for spatial cluster detection, the spatial scan statistics [1] have been extensively used in numerous applications including not only geographical disease surveillance but also architecture [2], forestry [3,4], astronomy [5], and criminology [6,7]. The method, based on a likelihood ratio test statistic, evaluates a large number of different and overlapping scanning windows. The test statistic is formulated based on a probability model depending on the data type, such as the Poisson model for count data [1] and the ordinal model for ordered categorical data [8]. Scanning windows are constructed with variable sizes at each location on a study area, up to a certain maximum limit. For each scanning window, a likelihood ratio test statistic for comparing inside versus outside the window is calculated and the scanning window with the maximum likelihood ratio is defined as the most likely cluster. The procedure of finding significant spatial clusters using the spatial scan statistics can be performed with the freely available software SaTScan™ [9].

An important issue regarding spatial scan statistics is the scanning window shape. The first proposed spatial scan statistic used circular-shaped scanning windows. The circular spatial scan statistic works well for compact clusters, but it may have difficulty correctly identifying non-circular clusters. Tango [10] and Tango and Takahashi [11] have mentioned that the original spatial scan statistic using circular windows tends to detect a larger cluster than the true cluster by swallowing neighboring areas with non-elevated risk. This phenomenon may occur more easily when the true cluster is non-circular. Other shapes of scanning windows also have been proposed such as elliptic [12] and irregular [11,13–17] shapes. Several studies [15–19] have shown that the methods using irregularly shaped scanning windows have a better power for detecting irregularly shaped clusters, as expected.

To apply the spatial scan statistics, one should determine the maximum scanning window size (MSWS) in advance. The MSWS is usually chosen in terms of the percentage of the total population for the study area, and an MSWS value of 50% of the total population is commonly used as the default setting for SaTScan™. However, users may choose an arbitrary MSWS and the results can be affected by the chosen MSWS. Ribeiro and Costa [20] examined the effect of different values of MSWS via a simulation study and found that the performance of spatial scan statistics can be sensitive to the choice of MSWS. Their findings do not imply that one may run the analysis multiple times with different values of MSWS to optimize the cluster detection results, as discussed by Han et al. [21]. In that case, the results will suffer from the multiple testing problem. Han et al. [21] proposed a method using a Gini coefficient to optimize the maximum reported cluster size (MRCS). It is statistically valid to rerun the analysis to report clusters of a certain maximum size while keeping the MSWS fixed at a larger value. Han et al. [21] mentioned that setting the MRCS at 50% often results in unnecessarily large and less informative clusters, and the authors concluded that the Gini coefficient can identify a more refined collection of non-overlapping clusters. This method has been implemented in SaTScan™ version 9.3.

In this paper, we have evaluated the use of the Gini coefficient in the spatial scan statistics for detecting irregularly shaped clusters. From our experience, we also found that using the Gini coefficient in SaTScan™ tends to result in the identification of multiple smaller clusters rather than a single larger cluster. The smaller clusters were often connected and located contiguously, in which case we may consider the clusters as a single cluster in a possibly irregular shape. We think that using the Gini coefficient improves the detection of irregularly shaped clusters. We do not expect that the use of the Gini coefficient outperforms other cluster detection methods specifically using irregularly shaped windows for the detection of irregular clusters. The Gini coefficient was developed as an optimizing criterion for MRCS, and if it has an ability to better detect irregularly shaped clusters than the original method, it certainly offers an advantage.

In the next section, we briefly review the spatial scan statistic for count data and the Gini coefficient in the Poisson-based scan statistic. Through a simulation study, we evaluate the performance of the Gini coefficient for detecting irregularly shaped clusters, compared with the original circular and elliptic scan statistics. Methods that were developed specifically for detecting irregular clusters, such as the flexible spatial scan statistic [11], the circular spatial scan statistic with a restricted likelihood ratio [22], and the flexible spatial scan statistic with a restricted likelihood ratio [23], are also included in the simulation study for comparison. We illustrate the different methods using a real data set of liver cancer mortality for males in Seoul and Gyeonggi province in Korea. Finally, we discuss our findings with some concluding remarks.

## Methods

### Spatial scan statistic for count data

When we want to detect a cluster of cases compared against the underlying population at risk, for example, using disease mortality data, we can use the Poisson-based spatial scan statistic. Given a collection of scanning windows *Z*, the spatial scan statistic for count data is defined as the maximum of the likelihood ratio test statistics over *Z* for the following hypotheses.

$$H_{0}:p=q\;for\;all\;z\in Z\;vs.\;H_{a}\;:\;p>q\;(or\;p<q)\;for\;some\;z\in Z$$

where *p* and *q* are the event rates inside and outside the scanning window *z*, respectively. The null hypothesis indicates no clustering and the alternative can be specified to search for clusters with high (or low) rates. The Poisson-based spatial scan statistic λ is expressed as

$$\lambda =\underset{z}{\max}\frac{L(z)}{L_{0}}=\underset{Z}{\max}\frac{{\left(\frac{c_{z}}{n_{z}}\right)}^{c_{z}}{\left(\frac{C-c_{z}}{N-n_{z}}\right)}^{C-c_{z}}}{{\left(\frac{C}{N}\right)}^{c}}I(\frac{c_{z}}{n_{z}}>\frac{C-c_{z}}{N-n_{z}})$$

where *c*_*z*_ and *n*_*z*_ denote the observed number of cases and the population within *z*, respectively, and *C* and *N* are the total number of observed cases and the total population over the whole area, respectively. I() is the indicator function to indicate a high or low rate. Because the denominator on the above formula does not depend on *z*, the term (*C*/*N*)^c^ often drops from the test statistic.

The most likely cluster is defined as the scanning window associated with the value of λ. The statistical significance of the most likely cluster is often assessed using Monte Carlo hypothesis testing, by generating random data sets under the null hypothesis and comparing the test statistic from the original data set with those from the randomly generated data sets. One may use Gumbel-based *p*-values by approximating the distribution of the test statistic to an extreme value distribution [24,25]. The two methods are available on SaTScan™.

Besides the most likely cluster, it can be informative to report secondary clusters with high likelihood ratios. The statistical significance of the secondary clusters is evaluated in the same way for the most likely cluster. As thoroughly explained in the paper by Han et al. [21], an earlier version of SaTScan™ reported secondary clusters without overlapping with more significant clusters as a default option, which could result in a large most likely cluster hiding several smaller distinct clusters. They proposed to apply the Gini coefficient as an intuitive and systematic way to determine the best collection of clusters to report by optimizing the MRCS. Here we briefly describe the method. The Gini coefficient for a set of clusters is calculated as two times the area between the reference line of *y* = *x* and the Lorenz curve. The Lorenz curve for a set of clusters is constructed using the cumulative percentages of observed cases and expected cases on the *x*- and *y*-axes, respectively. When there is a single significant cluster, as the number of observed cases in the cluster gets higher, which means more cases are concentrated, the Lorenz curve gets further away from the reference line and the Gini coefficient value gets higher. When comparing several competing collections of non-overlapping clusters, the one with the highest Gini coefficient value should be chosen as the cluster collection to report [21]. Through a simulation study, Han et al. [21] showed that the method identified the correct clusters and performed well. For more detailed information on the use of the Gini coefficient in the spatial scan statistic, refer to the paper by Han et al. [21]. The method has been implemented in SaTScan™ and is available for the Poisson and Bernoulli models only.

Although Han et al. [21] conducted a simulation study and showed a good performance of the Gini coefficient, they only considered compact clusters. Here we want to evaluate the Gini coefficient for detecting irregularly shaped clusters. As previously mentioned, if multiple small clusters of circular or elliptic shapes are found and they are located contiguously, they can be regarded as a single and possibly irregularly shaped cluster. We presumed that using the Gini coefficient can more precisely identify irregular clusters by reporting several smaller clusters connected to one another.

### Simulation study

**Fig 1:**
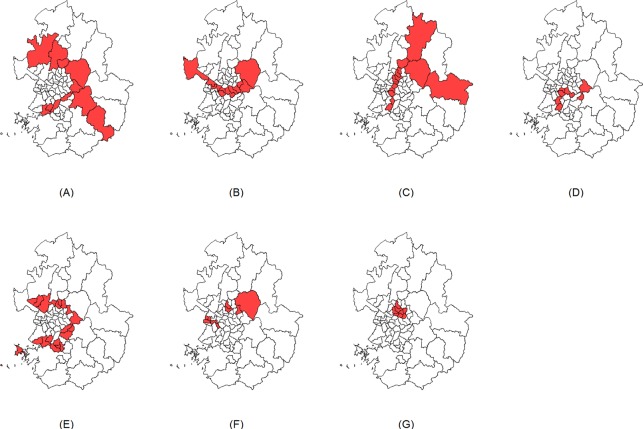
Simulated cluster models A–G.

We conducted an extensive simulation study to evaluate the performance of the Gini coefficient in the Poisson-based spatial scan statistic for detecting irregularly shaped clusters. We created 7 different cluster models with different shapes and at different locations on a real geographical map of Seoul and Gyeonggi province in South Korea. The area consists of 69 districts with mixed urban and rural regions. Seoul is the capital city of South Korea with a highly dense population and Gyeonggi province is composed of districts in relatively larger sizes with small populations. Table 1 and Fig 1 show the locations and information of the 7 simulated cluster models. We tried to create various types of cluster models in irregular shapes and in different locations and sizes. We also included a cluster model of a compact shape.

**Table 1:** Number of clusters and districts in the clusters of simulated cluster models A–G.

Cluster model	Number of clusters	Number of districts
A	1	11
B	1	12
C	1	14
D	2	2 / 5
E	2	7 / 11
F	3	2 / 2 / 4
G	1	8

We generated 1,000 random data sets for each cluster model with relative risks (RRs) of 1.3, 1.5, and 2 for the clusters of high rates. For the population for the study area, we used a half of the real population for each district of Seoul and Gyeonggi province in 2010 provided by Statistics Korea. For each randomly generated data set and each cluster model, we conducted a spatial cluster detection analysis using 7 different methods: the circular and elliptic spatial scan statistics with and without the Gini coefficient (denoted by CS, ES, GCS, and GES), the original flexible spatial scan statistic (OF), the circular spatial scan statistic with a restricted likelihood ratio (RC), and the flexible spatial scan statistic with a restricted likelihood ratio (RF). Analyses using CS, ES, GCS, and GES were conducted using SaTScan™ version 9.3 [9] and those using OF, RC, and RF were conducted using FleXScan version 3.1 [26].

We identified all significant clusters by each of the 7 methods for each simulation and calculated 3 performance measures, namely the usual power, sensitivity, and positive predictive power (PPV). The usual power indicates the power to reject the null hypothesis of no clustering (in any way) and was estimated by the number of rejections out of 1,000 replicate simulations. Tango and Takahashi [11] used the expression of the usual power, while they proposed a bivariate power to better reflect the accuracy of detecting true clusters. Sensitivity is defined as the number of districts correctly detected divided by the number of districts in the true cluster, and PPV is defined as the number of districts correctly detected divided by the number of detected districts. Sensitivity and PPV were estimated as the averages of sensitivity and PPV for data sets rejected at the 0.05 significance level.

We also estimated the bivariate power distribution proposed by Tango and Takahashi [11]. While the usual power, sensitivity, and PPV are useful for showing the performance as averaged measures, the bivariate power can reveal more detailed information on the accuracy of identifying the true cluster. The bivariate power distribution P(l,s) is defined with 2 parameters of length *l*, the number of regions of the detected cluster, and *s*, the number of regions identified correctly in the true cluster. The usual power can be obtained by summing up the bivariate power over all possible values of *l* and *s*. The bivariate power can indicate the probabilities of exact detection, under-detection, and over-detection.

### Korean male liver cancer mortality data

We analyzed Korean male liver cancer mortality data for 2010–2013 obtained from Statistics Korea. We used the aggregated mortality data at the “Si-Gun-Gu” (district) level and searched for clusters with high mortality rates in Seoul and Gyeonggi province using the 7 different methods. For the population, we used the 2010 Population and Housing Census data from Statistics Korea. The population and mortality data were grouped into 5-year age intervals and the analyses were adjusted for the age group.

## Results

### Simulation results

Tables 2**–**4 show the estimated usual power, sensitivity, and PPV for each method under cluster models A–G with the RRs of 1.3, 1.5, and 2, respectively. In most cases, the usual power was estimated as 1 or very close to 1. We included the usual power when it is not exactly equal to 1. Although none of the methods performed best across all scenarios, the RF method showed the highest values of sensitivity and PPV in many cases. The OF method performed relatively well overall. On the other hand, the CS and ES methods had poor performance in all scenarios except for scenario G of a compact cluster. ES performed very well and even better than the other methods for scenario G. The RC method showed very low values of sensitivity, especially when the relative risk was 1.3. The GES method performed reasonably well in general and better than CS, ES, and GCS did. The GCS method performed better than CS and ES overall, but ES seemed to perform better than GCS under some scenarios with RR = 1.3. The performance of GES was very comparable to those of RF and OF with even higher values of sensitivity or PPV in some scenarios. For a compact cluster, both the sensitivity and PPV of GES were higher than those of RF when the RR = 1.3 although PPV was somewhat lower when the RR = 1.5 or 2.

**Table 2:** Sensitivity and PPV for 7 cluster scenarios with RR = 1.3.

	CS	ES	GCS	GES	OF	RC	RF
**Scenario A**							
Sensitivity	0.543	0.661	0.546	0.690	0.676	0.450	0.698
PPV	0.793	0.904	0.794	0.906	0.830	0.968	0.876
Power	0.982	0.998	0.982	0.998	0.998	0.999	0.999
**Scenario B**							
Sensitivity	0.640	0.638	0.719	0.810	0.824	0.688	0.837
PPV	0.707	0.807	0.827	0.877	0.937	0.973	0.952
**Scenario C**							
Sensitivity	0.510	0.709	0.570	0.766	0.782	0.495	0.756
PPV	0.798	0.946	0.830	0.918	0.913	0.958	0.947
**Scenario D**							
Sensitivity	0.672	0.603	0.695	0.755	0.763	0.567	0.786
PPV	0.610	0.783	0.639	0.788	0.825	0.938	0.896
**Scenario E**							
Sensitivity	0.702	0.732	0.731	0.793	0.757	0.708	0.784
PPV	0.832	0.869	0.914	0.934	0.867	0.983	0.963
**Scenario F**							
Sensitivity	0.698	0.756	0.714	0.830	0.949	0.663	0.879
PPV	0.727	0.831	0.766	0.851	0.742	0.941	0.878
Power	0.999	0.999	0.999	0.999	1.000	0.998	1.000
**Scenario G**							
Sensitivity	0.871	0.960	0.873	0.959	0.945	0.832	0.942
PPV	0.958	0.988	0.945	0.979	0.958	0.989	0.961

The usual power = 1 for each method under scenarios B, C, D, and E. CS: Circular spatial scan statistic, ES: Elliptic spatial scan statistic, GCS: Circular spatial scan statistic using Gini coefficient, GES: Elliptic spatial scan statistic using Gini coefficient, OF: Flexible spatial scan statistic, RC: Circular spatial scan statistic with a restricted likelihood ratio. RF: Flexible spatial scan statistic with a restricted likelihood ratio.

**Table 3:** Sensitivity and PPV for 7 cluster scenarios with RR = 1.5.

	CS	ES	GCS	GES	OF	RC	RF
**Scenario A**							
Sensitivity	0.883	0.791	0.886	0.916	0.947	0.845	0.928
PPV	0.776	0.950	0.807	0.912	0.905	0.986	0.948
**Scenario B**							
Sensitivity	0.713	0.667	0.911	0.924	0.977	0.939	0.982
PPV	0.709	0.829	0.978	0.886	0.992	0.993	0.992
**Scenario C**							
Sensitivity	0.643	0.800	0.839	0.878	0.935	0.782	0.907
PPV	0.793	0.936	0.902	0.919	0.943	0.984	0.982
**Scenario D**							
Sensitivity	0.791	0.584	0.871	0.978	0.922	0.885	0.902
PPV	0.628	0.899	0.853	0.898	0.918	0.983	0.974
**Scenario E**							
Sensitivity	0.893	0.770	0.944	0.886	0.905	0.943	0.932
PPV	0.883	0.870	0.984	0.972	0.902	0.996	0.993
**Scenario F**							
Sensitivity	0.881	0.855	0.945	0.957	0.998	0.963	0.995
PPV	0.765	0.871	0.905	0.927	0.801	0.976	0.968
**Scenario G**							
Sensitivity	0.888	0.992	0.939	0.986	0.989	0.965	0.989
PPV	0.967	0.999	0.922	0.962	0.997	0.996	0.997

The usual power = 1 for each method under all scenarios. CS: Circular spatial scan statistic, ES: Elliptic spatial scan statistic, GCS: Circular spatial scan statistic using Gini coefficient, GES: Elliptic spatial scan statistic using Gini coefficient, OF: Flexible spatial scan statistic, RC: Circular spatial scan statistic with a restricted likelihood ratio. RF: Flexible spatial scan statistic with a restricted likelihood ratio.

**Table 4:** Sensitivity and PPV for 7 cluster scenarios with RR = 2.

	CS	ES	GCS	GES	OF	RC	RF
**Scenario A**							
Sensitivity	0.931	0.805	0.941	0.999	1.000	1.000	1.000
PPV	0.772	0.947	0.924	0.847	0.928	0.997	0.983
**Scenario B**							
Sensitivity	0.740	0.641	0.999	0.985	1.000	0.999	1.000
PPV	0.691	0.878	0.997	0.858	1.000	1.000	1.000
**Scenario C**							
Sensitivity	0.700	0.847	0.989	0.926	0.995	0.989	0.995
PPV	0.777	0.915	0.931	0.888	0.937	0.998	0.997
**Scenario D**							
Sensitivity	0.838	0.573	0.999	1.000	0.996	0.999	0.998
PPV	0.606	0.979	0.981	0.872	0.940	0.997	0.996
**Scenario E**							
Sensitivity	0.935	0.787	0.999	0.927	0.960	0.999	0.998
PPV	0.880	0.845	0.996	0.954	0.902	1.000	1.000
**Scenario F**							
Sensitivity	0.934	0.855	1.000	1.000	1.000	1.000	1.000
PPV	0.782	0.871	0.965	0.938	0.802	0.996	0.999
**Scenario G**							
Sensitivity	0.918	1.000	0.998	1.000	1.000	1.000	1.000
PPV	0.955	1.000	0.865	0.930	1.000	0.999	1.000

The usual power = 1 for each method under all scenarios. CS: Circular spatial scan statistic, ES: Elliptic spatial scan statistic, GCS: Circular spatial scan statistic using Gini coefficient, GES: Elliptic spatial scan statistic using Gini coefficient, OF: Flexible spatial scan statistic, RC: Circular spatial scan statistic with a restricted likelihood ratio. RF: Flexible spatial scan statistic with a restricted likelihood ratio.

The estimated bivariate power distribution for cluster model A with the RR of 1.5 for each method is shown in Table 5. Cluster model A represents a single irregularly shaped cluster composed of 11 districts. The usual power for each method was 1000/1000 as indicated in Table 3. The estimated probability of exact detection P(11,11) was highest for RF and RC. GES also had a relatively high value of exact detection probability compared to CS, ES, GCS, and OF. We observed that CS and GCS tended to over-detect (as represented by the large numbers presented in the rows greater than length *l* = 11), which led to a relatively low PPV (Table 3). RC seemed not to over-detect, but rather seemed to under-detect (as represented by the large numbers presented in the rows less than length *l* = 11), which led to a relatively low sensitivity. ES in this scenario also tended to under-detect. For OF and RF, larger numbers were distributed around the point of exact detection P(11,11). The bivariate power distribution for GES was comparable to that for OF or RF. Because the results of the bivariate power distribution for each method under all scenarios would take up too much space, here we only present 1 case as an example. Sensitivity and PPV provide enough information to compare the overall performance of the 7 methods. The results for the bivariate power distribution under all other scenarios can be found in S1 File.

**Table 5:** Estimated bivariate power distributions P(l,s) × 1,000 of the 7 methods for cluster model A (RR = 1.5).

	CS									ES							
	Included *s* hot-spot districts									Included *s* hot-spot districts							
*l*	5	6	7	8	9	10	11^#^	Total	*l*	5	6	7	8	9	10	11^#^	Total
5	2	0	0	0	0	0	0	2	5	1	0	0	0	0	0	0	1
6	0	5	0	0	0	0	0	5	6	0	7	0	0	0	0	0	7
7	0	0	20	0	0	0	0	20	7	0	1	63	0	0	0	0	64
8	0	0	0	24	0	0	0	24	8	0	0	11	409	0	0	0	420
9	0	0	3	1	6	0	0	10	9	0	0	0	111	41	0	0	152
10	0	1	2	14	0	0	0	17	10	0	0	2	15	46	71	0	134
11^#^	0	0	1	14	33	1	0	49	11^#^	0	0	0	5	14	17	52	88
12	0	0	0	1	170	8	0	179	12	0	0	0	0	3	7	79	89
13	0	0	0	0	2	522	1	525	13	0	0	0	0	2	4	3	9
14	0	0	0	0	1	7	114	122	14	0	0	0	0	1	2	5	8
15	0	0	0	0	2	1	1	4	15	0	0	0	0	2	0	0	2
16	0	0	0	1	1	8	2	12	16	0	0	0	0	0	9	10	19
17	0	0	0	0	0	5	24	29	17	0	0	0	0	0	3	3	6
18	0	0	0	0	0	0	2	2	18	0	0	0	0	0	0	1	1
Total*	2	6	26	55	215	552	144	1000	Total*	1	8	76	540	109	113	153	1000
	GCS									GES							
	Included *s* hot-spot districts									Included *s* hot-spot districts							
*l*	5	6	7	8	9	10	11^#^	Total	*l*	5	6	7	8	9	10	11^#^	Total
5	2	0	0	0	0	0	0	2	5	0	0	0	0	0	0	0	0
6	0	4	0	0	0	0	0	4	6	0	0	0	0	0	0	0	0
7	0	0	19	0	0	0	0	19	7	0	1	4	0	0	0	0	5
8	0	0	0	23	0	0	0	23	8	0	0	2	41	0	0	0	43
9	0	0	3	3	31	0	0	37	9	0	0	0	11	168	0	0	179
10	0	0	2	12	4	44	0	62	10	0	0	0	4	56	86	0	146
11^#^	0	0	1	7	61	4	7	80	11^#^	0	0	0	0	19	88	71	178
12	0	0	0	0	117	131	1	249	12	0	0	0	0	2	35	161	198
13	0	0	0	0	1	352	33	386	13	0	0	0	0	3	12	147	162
14	0	0	0	0	1	7	84	92	14	0	0	0	0	0	4	57	61
15	0	0	0	0	2	1	1	4	15	0	0	0	0	1	0	8	9
16	0	0	0	0	1	7	2	10	16	0	0	0	0	0	2	11	13
17	0	0	0	0	0	5	25	30	17	0	0	0	0	0	2	3	5
18	0	0	0	0	0	0	2	2	18	0	0	0	0	0	0	1	1
Total*	2	4	25	45	218	551	155	1000	Total*	0	1	6	56	249	229	459	1000
	OF									RC							
	Included *s* hot-spot districts									Included *s* hot-spot districts							
*l*	5	6	7	8	9	10	11^#^	Total	*l*	5	6	7	8	9	10	11^#^	Total
5	0	0	0	0	0	0	0	0	5	1	0	0	0	0	0	0	1
6	0	0	0	0	0	0	0	0	6	0	7	0	0	0	0	0	7
7	0	0	4	0	0	0	0	4	7	0	1	47	0	0	0	0	48
8	0	0	2	3	0	0	0	5	8	0	0	1	158	0	0	0	159
9	0	0	0	26	39	0	0	65	9	0	0	0	12	277	0	0	289
10	0	0	0	5	19	118	0	142	10	0	0	0	1	28	269	0	298
11^#^	0	0	0	2	5	148	17	172	11^#^	0	0	0	0	2	53	110	165
12	0	0	0	0	1	36	364	401	12	0	0	0	0	1	13	13	27
13	0	0	0	0	0	12	154	166	13	0	0	0	0	0	2	4	6
14	0	0	0	0	1	2	32	35	14	0	0	0	0	0	0	0	0
15	0	0	0	0	0	2	7	9	15	0	0	0	0	0	0	0	0
16	0	0	0	0	0	0	0	0	16	0	0	0	0	0	0	0	0
17	0	0	0	0	0	0	1	1	17	0	0	0	0	0	0	0	0
Total*	0	0	6	36	65	318	575	1000	Total*	1	8	48	171	308	337	127	1000
	RF																
	Included *s* hot-spot districts																
*l*	5	6	7	8	9	10	11^#^	Total									
5	0	0	0	0	0	0	0	0									
6	0	0	0	0	0	0	0	0									
7	0	0	4	0	0	0	0	4									
8	0	0	2	6	0	0	0	8									
9	0	0	0	7	60	0	0	67									
10	0	0	0	2	24	343	0	369									
11^#^	0	0	0	1	2	161	115	279									
12	0	0	0	0	0	30	162	192									
13	0	0	0	0	0	8	59	67									
14	0	0	0	0	1	1	10	12									
15	0	0	0	0	0	1	1	2									
Total*	0	0	6	16	87	544	347	1000									

CS: Circular spatial scan statistic, ES: Elliptic spatial scan statistic, GCS: Circular spatial scan statistic using Gini coefficient, GES: Elliptic spatial scan statistic using Gini coefficient, OF: Flexible spatial scan statistic, RC: Circular spatial scan statistic with a restricted likelihood ratio. RF: Flexible spatial scan statistic with a restricted likelihood ratio. 1000 trials were carried out.

*The usual power is 1000/1000.

^#^The number of districts in the true cluster for model A is 11.

### Analysis results for Korean male liver cancer mortality data

**Fig 2:**
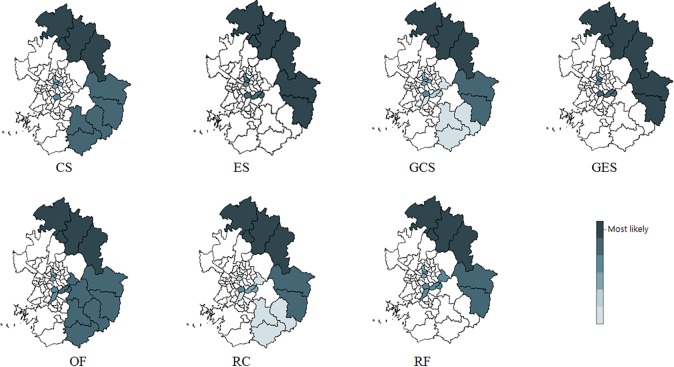
Spatial clusters with high mortality rates of male liver cancer in Seoul and Gyeonggi province in Korea for 2010–2013, detected by the 7 methods.

Fig 2 shows the detected clusters with high rates of Korean male liver cancer mortality in Seoul and Gyeonggi province for 2010–2013, using the 7 different methods. Table 6 includes information on the RR, *p*-value, and number of districts of the detected clusters. Overall, the results were similar for each method. On closer examination, however, the clusters detected by GES were almost identical to those detected by RF. The optimal MRCS for GES was found as small as 3% and still, ES (using 50% for MRCS) detected exactly the same clusters as GES. We think that this was due to the detected clusters having very high RRs and low populations. GES, and ES did not include only 2 small districts among the regions in the clusters detected by RF, while CS, GCS, OF, and RC identified more districts as significant clusters than RF. Although we do not know the true clusters in the real data, we assumed that the clusters detected by RF would be close to the true ones because the simulation studies in this paper and the paper by Tango [23] showed that RF has a very good performance for accurately identifying clusters.

**Table 6:** Most likely and secondary clusters of high rates of male liver cancer mortality in Seoul and Gyeonggi province in Korea for 2010–2013, detected by the 7 methods.

Cluster		CS	ES	GCS	GES	OF	RC	RF
1	RR	4.56	4.53	4.56	4.53	4.37	4.32	4.32
	*p*-value	<0.001	<0.001	<0.001	<0.001	<0.001	<0.001	<0.001
	# Districts	4	6	4	6	4	4	4
2	RR	2.00	2.18	4.02	2.18	1.89	3.92	3.92
	*p*-value	<0.001	<0.001	<0.001	<0.001	<0.001	<0.001	<0.001
	# Districts	6	3	2	3	9	2	2
3	RR	4.72	2.00	4.72	2.00	2.98	4.66	2.14
	*p*-value	<0.001	<0.001	<0.001	<0.001	<0.001	<0.001	<0.001
	# Districts	1	3	1	3	2	1	5
4	RR	1.64		1.64		1.95	1.77	1.95
	*p*-value	0.0042		<0.001		<0.001	0.003	<0.001
	# Districts	4		4		3	2	3
5	RR			1.8			1.84	
	*p*-value			<0.001			0.01	
	# Districts			2			2	
6	RR			1.61			1.59	
	*p*-value			0.012			0.015	
	# Districts			3			3	
7	RR			2.23			2.21	
	*p*-value			0.019			0.02	
	# Districts			1			1	
8	RR			2.21			2.21	
	*p*-value			0.029			0.025	
	# Districts			1			1	
9	RR						2.19	
	*p*-value						0.029	
	# Districts						1	

RR: Relative risk. CS: Circular spatial scan statistic, ES: Elliptic spatial scan statistic, GCS: Circular spatial scan statistic using Gini coefficient, GES: Elliptic spatial scan statistic using Gini coefficient, OF: Flexible spatial scan statistic, RC: Circular spatial scan statistic with a restricted likelihood ratio. RF: Flexible spatial scan statistic with a restricted likelihood ratio. Cluster 1 is the most likely cluster and the others are secondary by the order of statistical significance.

## Discussion

In this paper, we evaluated the use of the Gini coefficient in the Poisson-based spatial scan statistic for detecting irregularly shaped clusters. The simulation study showed that using the Gini coefficient in the elliptic spatial scan statistic had a reasonably good performance compared to the other methods for detecting irregular clusters. We think that the analysis results for Korean male liver cancer mortality data also support that the elliptic spatial scan statistic using the Gini coefficient might work well for detecting irregularly shaped clusters. Despite their popular usage in various applications, it has been pointed out that the spatial scan statistics with circular and elliptic shaped scanning windows may have difficulty in correctly identifying non-compact, arbitrarily shaped spatial clusters [13–19,22,23,27]. However, based on our simulation study, using the Gini coefficient in the elliptic spatial scan statistic can resolve the issue to a certain extent. We do not insist that the Gini coefficient can work better than other spatial cluster detection methods specifically using irregularly shaped windows for detecting arbitrarily shaped clusters. By reporting an optimized and refined collection of clusters, using the Gini coefficient can better identify irregularly shaped clusters than the original spatial scan statistic without using it. Also, its performance can be almost as good as the flexible spatial scan statistic with a restricted likelihood ratio. A major advantage of using the Gini coefficient over the flexible spatial scan statistic is efficiency in computation time. We found that running FleXScan with the RF method took two to three times longer than running SaTScan for our simulation study. Also, it could be a very tedious job to create a matrix definition file representing adjacency for each location, which is additionally required for FleXScan, for a data set having a very large number of locations.

The Gini coefficient has been already implemented in SaTScan™ for the Poisson and Bernoulli models. Spatial scan statistics are available for other probability models such as ordinal [8,28], multinomial [29], normal [30], and exponential [31]. It will be very useful to develop the Gini coefficient or another criterion for optimizing the MRCS for such models as well. While it is expected that such criterion measures may work well for detecting irregular clusters, a careful evaluation will be needed.

## Supporting Information

S1 File Results for bivariate power distributions. (PDF)
