# Supplementary material for: Evaluation of the Gini Coefficient in Spatial Scan Statistics for Detecting Irregularly Shaped Clusters
Source: PLoS One. 2017 Jan 27;12(1):e0170736. doi: 10.1371/journal.pone.0170736 (PMC5271318; doi:10.1371/journal.pone.0170736)
Supplement: S1 File — (PDF) [file pone.0170736.s001.pdf]

Supporting Information

Table A1. Estimated bivariate power distributions P(l,s) × 1,000 of the 7 methods for cluster model A (RR = 1.3).

	CS									ES							
	Included *s* hot-spot districts									Included *s* hot-spot districts							
l	1-5	6	7	8	9	10	11[#]	Total	*l*	1-5	6	7	8	9	10	11[#]	Total
1-5	313	0	0	0	0	0	0	313	1-5	132	0	0	0	0	0	0	132
6	18	43	0	0	0	0	0	61	6	14	72	0	0	0	0	0	86
7	35	8	8	0	0	0	0	51	7	4	17	137	0	0	0	0	158
8	17	26	1	1	0	0	0	45	8	2	8	58	147	0	0	0	215
9	7	60	13	0	0	0	0	80	9	0	6	11	84	23	0	0	124
10	3	10	100	24	0	0	0	137	10	1	1	11	34	42	24	0	113
11[#]	0	1	10	45	3	0	0	59	11[#]	0	1	1	18	12	18	10	60
12	3	3	1	5	91	0	0	103	12	1	1	3	10	1	4	15	35
13	1	1	7	6	8	30	0	53	13	0	3	0	3	4	4	7	21
14	0	1	4	3	3	3	0	14	14	0	1	1	4	2	2	2	12
15	0	2	0	5	4	8	0	19	15	0	0	0	0	8	3	1	12
16	0	0	0	0	3	15	1	19	16	0	1	0	0	4	9	0	14
17	0	1	0	0	2	4	4	11	17	0	1	0	0	1	2	1	5
18-30	1	2	1	2	4	5	2	17	18-30	0	1	2	0	3	4	1	11
Total*	398	158	145	91	118	65	7	982	Total*	154	113	224	300	100	70	37	998

	GCS									GES							
	Included *s* hot-spot districts									Included *s* hot-spot districts							
l	1-5	6	7	8	9	10	11[#]	Total	*l*	1-5	6	7	8	9	10	11[#]	Total
1-5	311	0	0	0	0	0	0	311	1-5	102	0	0	0	0	0	0	102
6	19	42	0	0	0	0	0	61	6	11	73	0	0	0	0	0	84
7	33	10	8	0	0	0	0	51	7	1	24	108	0	0	0	0	133
8	17	25	0	1	0	0	0	43	8	1	10	49	147	0	0	0	207
9	7	58	17	0	0	0	0	82	9	0	5	12	79	57	0	0	153
10	3	9	99	26	0	0	0	137	10	0	1	12	34	40	29	0	116
11[#]	0	1	9	43	5	0	0	58	11[#]	0	1	2	19	14	27	11	74
12	2	3	2	6	90	1	0	104	12	0	0	3	10	2	5	22	42
13	1	1	6	6	8	31	0	53	13	0	3	0	3	5	7	11	29
14	0	1	4	3	3	3	0	14	14	0	0	1	3	1	3	8	16
15	0	2	2	5	4	9	0	22	15	0	0	0	1	7	3	6	17
16	0	0	0	0	3	15	1	19	16	0	1	0	0	4	5	1	11
17	0	1	0	0	2	4	4	11	17	0	0	0	0	1	2	2	5
18-30	1	1	1	1	4	6	2	16	18-26	0	0	2	0	3	4	0	9
Total*	394	154	148	91	119	69	7	982	Total*	115	118	189	296	134	85	61	998

CS: Circular spatial scan statistic, ES: Elliptic spatial scan statistic, GCS: Circular spatial scan statistic using Gini coefficient, GES: Elliptic spatial scan statistic using Gini coefficient, OF: Flexible spatial scan statistic, RC: Circular spatial scan statistic with a restricted likelihood ratio. RF: Flexible spatial scan statistic with a restricted likelihood ratio. 1000 trials were carried out. *The usual power is 982/1000, 998/1000, 982/1000, and 998/1000 for CS, ES, GCS, and GES, respectively. [#]The number of districts in the true cluster for model A is 11.

Table A1. Estimated bivariate power distributions P(l,s) × 1,000 of the 7 methods for cluster model A (RR = 1.3) (continued).

	OF									RC							
	Included *s* hot-spot districts									Included *s* hot-spot districts							
l	1-5	6	7	8	9	10	11#	Total	*l*	1-5	6	7	8	9	10	11#	Total
1-5	116	0	0	0	0	0	0	116	1-5	557	0	0	0	0	0	0	557
6	25	27	0	0	0	0	0	52	6	35	173	0	0	0	0	0	208
7	15	19	83	0	0	0	0	117	7	11	28	97	0	0	0	0	136
8	3	3	75	1	0	0	0	82	8	0	11	20	24	0	0	0	55
9	1	4	47	105	7	0	0	164	9	0	1	8	8	4	0	0	21
10	1	4	32	94	12	15	0	158	10	0	0	3	5	2	0	0	10
11#	0	0	15	50	8	20	0	93	11#	0	0	0	2	4	0	0	6
12	0	0	11	25	7	20	20	83	12	0	0	0	0	1	0	0	1
13	0	0	4	7	2	13	17	43	13	0	0	0	0	0	0	0	0
14	0	0	4	8	4	11	21	48	14	0	0	0	0	0	0	0	0
15	0	0	0	1	1	4	11	17	15	0	0	0	0	0	0	0	0
16	0	0	0	2	0	1	6	9	16	0	0	0	0	0	0	0	0
17	0	0	0	0	1	3	6	10	17	0	0	0	0	0	0	0	0
18	0	0	0	0	1	2	3	6	18	0	0	0	0	0	0	0	0
Total*	161	57	271	293	43	89	84	998	Total*	603	213	128	39	11	0	0	994

	RF							
	Included *s* hot-spot districts							
l	1-5	6	7	8	9	10	11#	Total
1-5	86	0	0	0	0	0	0	86
6	18	43	0	0	0	0	0	61
7	4	28	143	0	0	0	0	175
8	2	10	97	12	0	0	0	121
9	0	4	64	58	29	0	0	155
10	0	3	22	39	27	45	0	136
11#	0	1	5	17	21	49	1	94
12	0	0	3	4	13	28	19	67
13	0	0	0	2	6	23	16	47
14	0	0	1	1	2	10	14	28
15	0	0	0	0	1	5	12	18
16	0	0	0	0	0	2	5	7
17	0	0	0	0	0	1	3	4
18	0	0	0	0	0	0	0	0
Total*	110	89	335	133	99	163	70	999

CS: Circular spatial scan statistic, ES: Elliptic spatial scan statistic, GCS: Circular spatial scan statistic using Gini coefficient, GES: Elliptic spatial scan statistic using Gini coefficient, OF: Flexible spatial scan statistic, RC: Circular spatial scan statistic with a restricted likelihood ratio. RF: Flexible spatial scan statistic with a restricted likelihood ratio. 1000 trials were carried out. *The usual power is 998/1000, 994/1000, and 999/1000 for OF, RC, and RF, respectively. #The number of districts in the true cluster for model A is 11.

Table A2. Estimated bivariate power distributions P(l,s) × 1,000 of the 7 methods for cluster model B (RR = 1.3).

	CS									ES							
	Included *s* hot-spot districts									Included *s* hot-spot districts							
l	1-6	7	8	9	10	11	12#	Total	*l*	1-6	7	8	9	10	11	12#	Total
1-5	27	0	0	0	0	0	0	27	1-5	25	0	0	0	0	0	0	25
6	35	0	0	0	0	0	0	35	6	100	0	0	0	0	0	0	100
7	55	26	0	0	0	0	0	81	7	6	197	0	0	0	0	0	203
8	43	21	1	0	0	0	0	65	8	1	177	8	0	0	0	0	186
9	58	21	32	0	0	0	0	111	9	2	66	12	13	0	0	0	93
10	2	17	52	5	0	0	0	76	10	0	14	9	18	0	0	0	41
11	4	33	37	21	0	0	0	95	11	0	1	12	6	9	0	0	28
12#	2	53	111	22	41	0	0	229	12#	0	1	47	10	10	5	0	73
13	1	8	20	42	7	9	0	87	13	0	1	9	4	8	0	0	22
14	0	4	30	7	19	3	1	64	14	0	0	5	32	0	2	0	39
15	0	6	3	12	5	3	0	29	15	0	0	5	6	2	1	0	14
16	0	0	1	3	0	0	0	4	16	0	0	9	4	2	2	0	17
17	0	0	4	13	4	0	0	21	17	0	1	3	8	0	0	1	13
18	0	0	7	0	15	0	0	22	18	0	0	0	33	3	1	0	37
19	0	0	0	1	3	2	0	6	19	0	0	0	39	30	0	0	69
20	0	1	0	0	2	0	0	3	20	0	0	0	3	7	0	0	10
21	0	0	1	0	0	0	0	1	21	0	0	0	1	3	1	0	5
22	0	0	0	1	0	1	0	2	22	0	0	0	1	4	0	0	5
23	0	1	0	1	0	0	0	2	23	0	0	0	0	1	0	2	3
24	0	0	0	0	0	0	0	0	24	0	0	0	0	1	9	0	10
25	0	0	0	1	0	0	0	1	25	0	0	0	0	0	5	0	5
26	0	0	0	19	1	0	0	20	26	0	0	0	0	1	0	0	1
27-29	0	0	3	5	11	0	0	19	27	0	0	0	0	0	1	0	1
Total*	227	191	302	153	108	18	1	1000	Total*	134	458	119	178	81	27	3	1000

	GCS									GES							
	Included *s* hot-spot districts									Included *s* hot-spot districts							
l	1-6	7	8	9	10	11	12#	Total	*l*	1-6	7	8	9	10	11	12#	Total
1-8	69	87	32	0	0	0	0	188	1-8	3	12	35	0	0	0	0	50
9	3	14	83	48	0	0	0	148	9	0	6	46	44	0	0	0	96
10	0	2	93	34	19	0	0	148	10	0	3	10	157	101	0	0	271
11	1	0	36	47	12	1	0	97	11	0	1	6	50	143	1	0	201
12#	0	1	23	38	141	0	0	203	12#	0	0	1	11	53	131	0	196
13	0	1	5	25	23	36	0	90	13	0	0	0	6	21	42	0	69
14	0	0	11	7	21	11	2	52	14	0	0	0	1	6	24	18	49
15	0	4	4	11	9	4	0	32	15	0	1	1	2	4	9	4	21
16	0	0	0	6	2	0	0	8	16	0	0	0	1	1	8	2	12
17	0	0	1	1	4	0	0	6	17	0	0	0	1	0	0	2	3
18	0	0	0	0	11	0	0	11	18	0	0	0	3	8	1	0	12
19	0	0	2	0	0	5	0	7	19	0	0	0	3	4	0	0	7
20	0	0	0	0	1	1	0	2	20	0	0	0	1	1	1	0	3
21	0	0	0	0	0	0	0	0	21	0	0	0	0	1	1	0	2
22	0	0	0	0	0	2	0	2	22	0	0	0	0	0	0	1	1
23-29	0	0	0	2	4	0	0	6	23-25	0	0	0	0	0	6	1	7
Total*	73	109	290	219	247	60	2	1000	Total*	3	23	99	280	343	224	28	1000

CS: Circular spatial scan statistic, ES: Elliptic spatial scan statistic, GCS: Circular spatial scan statistic using Gini coefficient, GES: Elliptic spatial scan statistic using Gini coefficient, OF: Flexible spatial scan statistic, RC: Circular spatial scan statistic with a restricted likelihood ratio. RF: Flexible spatial scan statistic with a restricted likelihood ratio. 1000 trials were carried out. *The usual power is 1000/1000. #The number of districts in the true cluster for model B is 12.

Table A2. Estimated bivariate power distributions P(l,s) × 1,000 of the 7 methods for cluster model B (RR = 1.3) (continued).

	OF									RC							
	Included *s* hot-spot districts									Included *s* hot-spot districts							
l	1-6	7	8	9	10	11	12#	Total	*l*	6	7	8	9	10	11	12#	Total
1-8	19	5	45	0	0	0	0	69	8	94	199	202	0	0	0	0	495
9	1	1	12	179	0	0	0	193	9	0	2	61	193	0	0	0	256
10	0	0	7	96	137	0	0	240	10	0	0	3	40	106	0	0	149
11	0	0	1	39	103	66	0	209	11	0	0	1	9	37	27	0	74
12#	0	0	2	19	30	28	72	151	12#	0	0	0	1	6	11	1	19
13	0	0	0	4	12	16	55	87	13	0	0	0	0	2	4	1	7
14	0	0	0	0	0	5	24	29	14	0	0	0	0	0	0	0	0
15-18	0	0	0	0	2	3	17	22	15	0	0	0	0	0	0	0	0
Total*	20	6	67	337	284	118	168	1000	Total*	94	201	267	243	151	42	2	1000

	RF							
	Included *s* hot-spot districts							
l	6	7	8	9	10	11	12#	Total
8	12	5	46	0	0	0	0	63
9	1	0	10	190	0	0	0	201
10	0	0	6	77	156	0	0	239
11	0	0	1	38	87	81	0	207
12#	0	0	1	11	16	32	109	169
13	0	0	0	1	1	11	70	83
14	0	0	0	0	0	6	32	38
Total*	13	5	64	317	260	130	211	1000

CS: Circular spatial scan statistic, ES: Elliptic spatial scan statistic, GCS: Circular spatial scan statistic using Gini coefficient, GES: Elliptic spatial scan statistic using Gini coefficient, OF: Flexible spatial scan statistic, RC: Circular spatial scan statistic with a restricted likelihood ratio. RF: Flexible spatial scan statistic with a restricted likelihood ratio. 1000 trials were carried out. *The usual power is 1000/1000. #The number of districts in the true cluster for model B is 12.

Table A3. Estimated bivariate power distributions P(l,s) × 1,000 of the 7 methods for cluster model C (RR = 1.3).

	CS								ES							
	Included *s* hot-spot districts									Included *s* hot-spot districts						
l	1-6	7-8	9-10	11	12	13	$14^{\#}$	Total	*l*	5-8	9	10	11	12	13	$14^{\#}$	Total
1-5	162	0	0	0	0	0	0	162	1-5	1	0	0	0	0	0	0	1
6	104	0	0	0	0	0	0	104	6	0	0	0	0	0	0	0	0
7	73	31	0	0	0	0	0	104	7	14	0	0	0	0	0	0	14
8	24	33	0	0	0	0	0	57	8	13	0	0	0	0	0	0	13
9	91	50	3	0	0	0	0	144	9	17	446	0	0	0	0	0	463
10	7	57	2	0	0	0	0	66	10	2	43	10	0	0	0	0	55
11	1	47	38	0	0	0	0	86	11	1	3	115	39	0	0	0	158
12	0	24	39	0	0	0	0	63	12	0	5	3	23	44	0	0	75
13	1	15	29	11	0	0	0	56	13	0	1	6	15	92	0	0	114
$14^{\#}$	0	11	18	4	0	0	0	33	$14^{\#}$	0	1	3	33	36	0	0	73
15	1	6	35	6	0	0	0	48	15	0	0	0	5	5	8	0	18
16	0	3	10	2	1	0	0	16	16	0	0	0	0	4	3	0	7
17	0	4	7	5	4	0	0	20	17	0	0	0	0	2	2	0	4
18	0	2	0	0	2	6	0	10	18	0	0	0	0	0	1	2	3
19	0	2	4	0	1	2	0	9	19	0	0	0	0	0	2	0	2
20	0	0	3	0	2	1	0	6	20	0	0	0	0	0	0	0	0
21-26	0	0	8	3	3	2	0	16	21	0	0	0	0	0	0	0	0
Total*	464	285	196	31	13	11	0	1000	Total*	48	499	137	115	183	16	2	1000

	GCS								GES							
	Included *s* hot-spot districts									Included *s* hot-spot districts						
l	1-8	9	10	11	12	13	$14^{\#}$	Total	*l*	8	9	10	11	12	13	$14^{\#}$	Total
1-7	263	0	0	0	0	0	0	263	7	10	0	0	0	0	0	0	10
8	104	0	0	0	0	0	0	104	8	22	0	0	0	0	0	0	22
9	92	33	0	0	0	0	0	125	9	17	110	0	0	0	0	0	127
10	67	13	7	0	0	0	0	87	10	6	46	80	0	0	0	0	132
11	33	65	4	0	0	0	0	102	11	2	14	64	54	0	0	0	134
12	18	32	47	3	0	0	0	100	12	0	4	33	77	84	0	0	198
13	7	12	24	15	1	0	0	59	13	0	0	11	37	138	4	0	190
$14^{\#}$	6	13	12	10	3	0	0	44	$14^{\#}$	0	1	3	22	57	16	1	100
15	4	4	25	10	1	0	0	44	15	0	0	1	6	16	21	0	44
16	2	5	3	4	4	0	0	18	16	0	0	0	1	9	9	5	24
17	3	3	4	5	5	0	0	20	17	0	0	0	2	2	4	0	8
18	0	0	0	1	2	8	0	11	18	0	0	0	0	1	3	2	6
19	0	2	0	0	1	2	0	5	19	0	0	0	0	0	3	1	4
20	0	1	7	2	5	3	0	18	20	0	0	0	0	0	0	1	1
Total*	599	183	133	50	22	13	0	1000	Total*	57	175	192	199	307	60	10	1000

CS: Circular spatial scan statistic, ES: Elliptic spatial scan statistic, GCS: Circular spatial scan statistic using Gini coefficient, GES: Elliptic spatial scan statistic using Gini coefficient, OF: Flexible spatial scan statistic, RC: Circular spatial scan statistic with a restricted likelihood ratio. RF: Flexible spatial scan statistic with a restricted likelihood ratio. 1000 trials were carried out. *The usual power is 1000/1000. #The number of districts in the true cluster for model C is 14.

Table A3. Estimated bivariate power distributions P(l,s) × 1,000 of the 7 methods for cluster model C (RR = 1.3) (continued).

	OF									RC							
	Included *s* hot-spot districts									Included *s* hot-spot districts							
l	1-8	9	10	11	12	13	14#	Total	*l*	1-8	9	10	11	12	13	14#	Total
1-6	9	0	0	0	0	0	0	9	1-6	337	0	0	0	0	0	0	337
7	22	0	0	0	0	0	0	22	7	249	0	0	0	0	0	0	249
8	6	0	0	0	0	0	0	6	8	193	0	0	0	0	0	0	193
9	1	29	0	0	0	0	0	30	9	68	43	0	0	0	0	0	111
10	2	14	108	0	0	0	0	124	10	18	29	18	0	0	0	0	65
11	0	11	79	123	0	0	0	213	11	3	15	7	1	0	0	0	26
12	1	1	48	121	17	0	0	188	12	0	2	6	5	0	0	0	13
13	0	2	19	68	93	6	0	188	13	0	0	5	1	0	0	0	6
14#	0	0	9	16	33	25	0	83	14#	0	0	0	0	0	0	0	0
15	0	0	1	9	13	16	38	77	15	0	0	0	0	0	0	0	0
16	0	0	0	4	2	14	21	41	16	0	0	0	0	0	0	0	0
17	0	0	0	1	0	3	15	19	17	0	0	0	0	0	0	0	0
Total*	41	57	264	342	158	64	74	1000		868	89	36	7	0	0	0	1000

	RF							
	Included *s* hot-spot districts							
l	8	9	10	11	12	13	14#	Total
10	52	61	0	0	0	0	0	113
11	1	25	181	0	0	0	0	207
12	0	8	81	183	0	0	0	272
13	0	2	32	104	49	0	0	187
14#	1	0	14	48	41	25	0	129
15	0	0	2	7	20	26	1	56
16	0	0	0	1	6	10	9	26
Total*	0	0	0	0	1	3	6	1000

CS: Circular spatial scan statistic, ES: Elliptic spatial scan statistic, GCS: Circular spatial scan statistic using Gini coefficient, GES: Elliptic spatial scan statistic using Gini coefficient, OF: Flexible spatial scan statistic, RC: Circular spatial scan statistic with a restricted likelihood ratio. RF: Flexible spatial scan statistic with a restricted likelihood ratio. 1000 trials were carried out. *The usual power is 1000/1000. #The number of districts in the true cluster for model C is 14.

Table A4. Estimated bivariate power distributions P(l,s) × 1,000 of the 7 methods for cluster model D (RR = 1.3).

	CS									ES							
	Included *s* hot-spot districts									Included *s* hot-spot districts							
l	1	2	3	4	5	6	$7^{\#}$	Total	*l*	1	2	3	4	5	6	$7^{\#}$	Total
1-2	0	68	0	0	0	0	0	68	1-3	0	13	54	0	0	0	0	67
3	0	7	45	0	0	0	0	52	4	0	0	81	380	0	0	0	461
4	0	1	10	2	0	0	0	13	5	0	0	8	5	7	0	0	20
5	0	1	27	51	0	0	0	79	6	0	0	1	119	22	7	0	149
6	0	0	9	27	8	0	0	44	$7^{\#}$	0	0	0	84	10	2	0	96
$7^{\#}$	0	0	5	194	2	0	0	201	8	0	0	0	11	5	6	0	22
8	0	0	1	14	119	1	0	135	9	0	0	0	9	29	8	0	46
9	0	0	0	5	18	7	0	30	10	0	0	0	1	20	4	0	25
10	0	0	0	2	7	170	0	179	11	0	0	0	0	5	4	3	12
11	0	0	0	3	16	20	2	41	12	0	0	0	0	8	1	1	10
12	0	0	0	0	3	16	1	20	13	0	0	0	0	3	2	0	5
13	0	0	0	0	0	5	0	5	14	0	0	0	0	1	22	2	25
14	0	0	0	0	0	1	14	15	15	0	0	0	0	0	9	0	9
15	0	0	0	0	0	5	3	8	16	0	0	0	0	0	7	12	19
16-34	0	0	0	0	10	36	64	110	17	0	0	0	0	1	15	18	34
Total*	0	77	97	298	183	261	84	1000	Total*	0	13	144	609	111	87	36	1000

	GCS									GES							
	Included *s* hot-spot districts									Included *s* hot-spot districts							
l	1	2	3	4	5	6	$7^{\#}$	Total	*l*	1	2	3	4	5	6	$7^{\#}$	Total
1-4	0	48	53	30	0	0	0	131	1-4	0	5	31	82	0	0	0	118
5	0	0	20	41	11	0	0	72	5	0	0	1	44	95	0	0	140
6	0	0	8	53	25	3	0	89	6	0	0	0	32	155	62	0	249
$7^{\#}$	0	0	5	119	29	12	0	165	$7^{\#}$	0	0	0	31	48	60	50	189
8	0	0	1	6	120	11	0	138	8	0	0	0	3	19	26	28	76
9	0	0	0	5	32	19	0	56	9	0	0	0	3	19	50	3	75
10	0	0	0	3	9	146	4	162	10	0	0	0	0	11	10	5	26
11	0	0	0	3	12	25	6	46	11	0	0	0	0	3	8	14	25
12	0	0	0	0	3	20	3	26	12	0	0	0	0	4	6	10	20
13	0	0	0	0	1	8	0	9	13	0	0	0	0	1	5	5	11
14	0	0	0	0	0	3	15	18	14	0	0	0	0	0	16	4	20
15	0	0	0	0	0	5	4	9	15	0	0	0	0	0	9	2	11
16-34	0	0	0	0	3	25	51	79	16	0	0	0	0	1	16	23	40
Total*	0	48	87	260	245	277	83	1000	Total*	0	5	32	195	356	268	144	1000

CS: Circular spatial scan statistic, ES: Elliptic spatial scan statistic, GCS: Circular spatial scan statistic using Gini coefficient, GES: Elliptic spatial scan statistic using Gini coefficient, OF: Flexible spatial scan statistic, RC: Circular spatial scan statistic with a restricted likelihood ratio. RF: Flexible spatial scan statistic with a restricted likelihood ratio. 1000 trials were carried out. *The usual power is 1000/1000. #The number of districts in the true cluster for model D is 7.

Table A4. Estimated bivariate power distributions P(l,s) × 1,000 of the 7 methods for cluster model D (RR = 1.3) (continued).

	OF							
	Included *s* hot-spot districts							
l	1	2	3	4	5	6	$7^{\#}$	Total
1-4	0	1	12	118	0	0	0	131
5	0	0	3	114	17	0	0	134
6	0	0	2	52	88	128	0	270
$7^{\#}$	0	0	0	17	54	80	0	151
8	0	0	0	10	14	25	100	149
9	0	0	0	4	6	21	56	87
10	0	0	0	2	1	12	19	34
11	0	0	0	0	1	7	20	28
12	0	0	0	0	0	2	8	10
13	0	0	0	0	0	0	6	6
Total*	0	1	17	317	181	275	209	1000

	RC							
	Included *s* hot-spot districts							
l	1	2	3	4	5	6	$7^{\#}$	Total
1-4	1	62	262	273	0	0	0	598
5	0	0	5	65	146	0	0	216
6	0	0	1	23	68	27	0	119
$7^{\#}$	0	0	0	5	22	20	0	47
8	0	0	0	0	4	11	1	16
9	0	0	0	0	0	4	0	4
10	0	0	0	0	0	0	0	0
11	0	0	0	0	0	0	0	0
12	0	0	0	0	0	0	0	0
13	0	0	0	0	0	0	0	0
Total*	1	62	268	366	240	62	1	1000

	RF							
	Included *s* hot-spot districts							
l	1	2	3	4	5	6	$7^{\#}$	Total
1-4	0	1	9	146	0	0	0	156
5	0	0	3	61	52	0	0	116
6	0	0	0	18	29	306	0	353
$7^{\#}$	0	0	0	2	18	143	2	165
8	0	0	1	1	7	49	53	111
9	0	0	0	0	3	38	58	99
Total*	0	1	13	228	109	536	113	1000

CS: Circular spatial scan statistic, ES: Elliptic spatial scan statistic, GCS: Circular spatial scan statistic using Gini coefficient, GES: Elliptic spatial scan statistic using Gini coefficient, OF: Flexible spatial scan statistic, RC: Circular spatial scan statistic with a restricted likelihood ratio. RF: Flexible spatial scan statistic with a restricted likelihood ratio. 1000 trials were carried out. *The usual power is 1000/1000. $^{\#}$The number of districts in the true cluster for model D is 7.

Table A5. Estimated bivariate power distributions P(l,s) × 1,000 of the 7 methods for cluster model E (RR = 1.3).

CS								
	Included *s* hot-spot districts							
l	1-12	13	14	15	16	17	18#	Total
1-11	150	0	0	0	0	0	0	150
12	67	0	0	0	0	0	0	67
13	100	13	0	0	0	0	0	113
14	53	37	8	0	0	0	0	98
15	24	44	13	8	0	0	0	89
16	22	35	26	47	1	0	0	131
17	8	10	17	6	12	0	0	53
18#	8	9	20	19	23	2	0	81
19	11	4	9	7	19	7	0	57
20	5	5	1	1	3	0	0	15
21	6	34	3	1	5	10	0	59
22	1	2	10	5	1	0	4	23
23	2	0	2	4	1	0	0	9
24	1	3	3	3	17	0	0	27
25	0	0	0	1	5	2	1	9
26	0	2	2	1	2	2	0	9
27	0	0	0	1	1	1	0	3
28	0	0	0	1	1	2	0	4
29-32	0	0	0	0	2	1	0	3
Total*	458	198	114	105	93	27	5	1000

ES								
	Included *s* hot-spot districts							
l	1-12	13	14	15	16	17	18#	Total
1-10	57	0	0	0	0	0	0	57
11	73	0	0	0	0	0	0	73
12	77	0	0	0	0	0	0	77
13	79	13	0	0	0	0	0	92
14	31	29	13	0	0	0	0	73
15	15	108	11	21	0	0	0	155
16	8	40	28	7	12	0	0	95
17	3	6	89	10	8	1	0	117
18#	1	5	20	67	3	6	0	102
19	0	0	8	20	13	3	0	44
20	1	1	10	23	31	1	1	68
21	0	0	1	4	15	0	0	20
22	0	1	0	0	3	4	0	8
23	0	0	1	0	3	5	0	9
24	0	0	0	0	2	0	0	2
25	0	0	0	0	2	3	0	5
26	0	0	0	1	0	0	0	1
27	0	0	0	0	0	0	0	0
28	0	0	0	0	1	1	0	2
Total*	345	203	181	153	93	24	1	1000

GCS								
	Included *s* hot-spot districts							
l	1-12	13	14	15	16	17	18#	Total
1-13	302	86	0	0	0	0	0	388
14	33	44	66	0	0	0	0	143
15	14	35	26	41	0	0	0	116
16	5	19	15	34	55	0	0	128
17	3	7	13	10	6	22	0	61
18#	3	4	15	9	20	3	2	56
19	4	0	2	4	16	5	1	32
20	0	3	1	1	4	1	0	10
21	1	5	1	1	4	12	0	24
22	2	1	5	0	1	1	3	13
23	0	0	0	3	1	0	0	4
24	0	1	1	2	6	0	0	10
25	0	0	0	2	3	0	1	6
26	0	0	1	0	1	1	1	4
27	0	0	0	0	3	2	0	5
Total*	367	205	146	107	120	47	8	1000

GES								
	Included *s* hot-spot districts							
l	1-12	13	14	15	16	17	18#	Total
1-13	121	51	0	0	0	0	0	172
14	14	34	118	0	0	0	0	166
15	7	33	48	153	0	0	0	241
16	1	15	21	52	60	0	0	149
17	1	6	24	12	48	18	0	109
18#	0	2	6	27	18	26	2	81
19	0	0	0	4	6	14	1	25
20	0	0	4	10	12	2	1	29
21	0	0	1	1	8	1	0	11
22	0	0	0	0	1	2	0	3
23	0	0	1	0	2	2	0	5
24	0	0	0	0	2	0	0	2
25	0	0	0	0	2	3	0	5
26	0	0	0	1	0	0	0	1
27-28	0	0	0	0	1	0	0	1
Total*	144	141	223	260	160	68	4	1000

CS: Circular spatial scan statistic, ES: Elliptic spatial scan statistic, GCS: Circular spatial scan statistic using Gini coefficient, GES: Elliptic spatial scan statistic using Gini coefficient, OF: Flexible spatial scan statistic, RC: Circular spatial scan statistic with a restricted likelihood ratio. RF: Flexible spatial scan statistic with a restricted likelihood ratio. 1000 trials were carried out. *The usual power is 1000/1000. #The number of districts in the true cluster for model E is 18.

Table A5. Estimated bivariate power distributions P(l,s) × 1,000 of the 7 methods for cluster model E (RR = 1.3) (continued).

	OF									RC							
	Included *s* hot-spot districts									Included *s* hot-spot districts							
l	1-12	13	14	15	16	17	18#	Total	*l*	1-12	13	14	15	16	17	18#	Total
1-13	138	0	0	0	0	0	0	138	1-13	426	143	0	0	0	0	0	569
14	89	72	4	0	0	0	0	165	14	3	40	124	0	0	0	0	167
15	40	131	10	5	0	0	0	186	15	0	1	25	81	0	0	0	107
16	10	63	85	23	7	0	0	188	16	0	0	5	26	63	0	0	94
17	3	18	42	28	25	0	0	116	17	0	0	0	5	14	30	0	49
18#	1	2	16	11	37	10	0	77	18*	0	0	0	0	1	4	3	8
19	0	0	4	7	24	35	0	70	19	0	0	0	0	0	5	1	6
20	0	0	0	2	6	19	12	39	20	0	0	0	0	0	0	0	0
21	0	0	0	0	3	5	5	13	21	0	0	0	0	0	0	0	0
22-25	0	0	0	0	1	5	2	8	22	0	0	0	0	0	0	0	0
Total*	281	286	161	76	103	74	19	1000	Total*	429	184	154	112	78	39	4	1000

	RF							
	Included *s* hot-spot districts							
l	1-12	13	14	15	16	17	18#	Total
1-13	184	50	0	0	0	0	0	234
14	14	42	105	0	0	0	0	161
15	0	11	54	172	0	0	0	237
16	1	3	17	71	119	0	0	211
17	1	1	4	23	68	12	0	109
18#	0	0	0	3	14	11	8	36
19	0	0	0	0	3	4	3	10
20	0	0	0	0	0	1	1	2
Total*	200	107	180	269	204	28	12	1000

CS: Circular spatial scan statistic, ES: Elliptic spatial scan statistic, GCS: Circular spatial scan statistic using Gini coefficient, GES: Elliptic spatial scan statistic using Gini coefficient, OF: Flexible spatial scan statistic, RC: Circular spatial scan statistic with a restricted likelihood ratio. RF: Flexible spatial scan statistic with a restricted likelihood ratio. 1000 trials were carried out. *The usual power is 1000/1000. #The number of districts in the true cluster for model E is 18.

Table A6. Estimated bivariate power distributions P(l,s) × 1,000 of the 7 methods for cluster model F (RR = 1.3).

	CS									ES							
	Included *s* hot-spot districts									Included *s* hot-spot districts							
l	1-2	3	4	5	6	7	8[#]	Total	*l*	1-2	3	4	5	6	7	8[#]	Total
1-4	32	39	26	0	0	0	0	97	1-4	18	20	32	0	0	0	0	70
5	1	1	60	20	0	0	0	82	5	0	0	9	150	0	0	0	159
6	0	2	9	136	6	0	0	153	6	0	0	1	46	77	0	0	124
7	0	0	10	27	136	0	0	173	7	0	0	2	17	162	37	0	218
8[#]	0	1	4	21	35	13	0	74	8[#]	0	0	1	8	47	75	19	150
9	0	1	0	9	21	81	0	112	9	0	0	0	1	12	57	22	92
10	0	1	3	3	50	39	12	108	10	0	0	1	0	4	19	16	40
11	0	0	0	4	11	20	5	40	11	0	0	0	0	0	27	10	37
12	0	0	0	2	8	5	9	24	12	0	0	0	1	0	6	19	26
13	0	0	1	0	25	8	5	39	13	0	0	0	2	0	3	11	16
14	0	0	1	1	3	18	3	26	14	0	0	1	2	1	0	8	12
15	0	0	1	1	2	5	1	10	15	0	0	0	0	0	2	1	3
16	0	0	0	0	2	5	0	7	16	0	0	0	0	0	1	1	2
17	0	0	0	0	1	3	2	6	17	0	0	0	0	2	1	3	6
18	0	0	0	1	0	1	0	2	18	0	0	0	0	0	1	0	1
19	0	0	2	1	3	2	2	10	19	0	0	0	0	2	0	1	3
20	0	0	0	1	0	6	1	8	20	0	0	0	0	1	0	0	1
21-29	0	0	0	0	5	11	12	28	21-29	0	0	0	0	2	13	24	39
Total*	33	45	117	227	308	217	52	999	Total*	18	20	47	227	310	242	135	999

	GCS									GES							
	Included *s* hot-spot districts									Included *s* hot-spot districts							
l	1-2	3	4	5	6	7	8[#]	Total	*l*	1-2	3	4	5	6	7	8[#]	Total
1-4	30	39	40	0	0	0	0	109	1-4	8	8	17	0	0	0	0	33
5	0	1	44	45	0	0	0	90	5	0	0	8	90	0	0	0	98
6	0	2	6	112	31	0	0	151	6	0	0	1	18	72	0	0	91
7	0	0	6	25	118	11	0	160	7	0	0	1	11	86	112	0	210
8[#]	0	0	3	13	44	32	3	95	8[#]	0	0	0	3	39	83	73	198
9	0	1	0	9	21	92	6	129	9	0	0	0	1	14	55	78	148
10	0	1	1	3	27	43	17	92	10	0	0	0	1	4	27	41	73
11	0	0	0	2	12	20	6	40	11	0	0	0	0	1	29	20	50
12	0	0	0	0	6	8	10	24	12	0	0	0	0	0	8	26	34
13	0	0	1	0	10	7	5	23	13	0	0	0	1	0	6	12	19
14	0	0	1	1	2	18	4	26	14	0	0	1	1	0	3	11	16
15	0	0	1	1	3	4	3	12	15	0	0	0	0	0	3	2	5
16	0	0	0	0	3	3	1	7	16	0	0	0	0	0	1	2	3
17	0	0	0	0	1	2	2	5	17	0	0	0	0	0	2	4	6
18-29	0	0	1	3	2	14	16	36	18-29	0	0	0	0	0	5	10	15
Total*	30	44	104	214	280	254	73	999	Total*	8	8	28	126	216	334	279	999

CS: Circular spatial scan statistic, ES: Elliptic spatial scan statistic, GCS: Circular spatial scan statistic using Gini coefficient, GES: Elliptic spatial scan statistic using Gini coefficient, OF: Flexible spatial scan statistic, RC: Circular spatial scan statistic with a restricted likelihood ratio. RF: Flexible spatial scan statistic with a restricted likelihood ratio. 1000 trials were carried out. *The usual power is 999/1000. [#]The number of districts in the true cluster for model F is 8.

Table A6. Estimated bivariate power distributions P(l,s) × 1,000 of the 7 methods for cluster model F (RR = 1.3) (continued).

	OF									RC							
	Included *s* hot-spot districts									Included *s* hot-spot districts							
l	1-2	3	4	5	6	7	8[#]	Total	*l*	1-2	3	4	5	6	7	8[#]	Total
1-6	1	0	17	4	17	0	0	39	1-6	25	86	138	250	174	0	0	673
7	0	0	8	0	5	0	0	13	7	0	0	0	17	97	76	0	190
8[#]	0	0	5	2	12	26	0	45	8[#]	0	0	1	1	14	71	7	94
9	0	0	4	1	6	50	85	146	9	0	0	0	0	2	17	14	33
10	0	0	1	1	3	38	239	282	10	0	0	0	0	0	1	5	6
11	0	0	1	0	1	15	225	242	11	0	0	0	0	0	0	2	2
12	0	0	0	0	0	11	133	144	12	0	0	0	0	0	0	0	0
13-20	0	0	0	0	2	4	83	89	13	0	0	0	0	0	0	0	0
Total*	1	0	36	8	46	144	765	1000	Total*	25	86	139	268	287	165	28	998

	RF							
	Included *s* hot-spot districts							
l	1-2	3	4	5	6	7	8*	Total
1-6	5	6	42	36	118	0	0	207
7	0	0	0	1	63	47	0	111
8[#]	0	0	2	2	37	55	121	217
9	0	0	0	1	13	29	209	252
10	0	0	0	0	1	12	111	124
11	0	0	0	0	1	4	57	62
12-15	0	0	0	0	1	1	25	27
Total*	5	6	44	40	234	148	523	1000

CS: Circular spatial scan statistic, ES: Elliptic spatial scan statistic, GCS: Circular spatial scan statistic using Gini coefficient, GES: Elliptic spatial scan statistic using Gini coefficient, OF: Flexible spatial scan statistic, RC: Circular spatial scan statistic with a restricted likelihood ratio. RF: Flexible spatial scan statistic with a restricted likelihood ratio. 1000 trials were carried out. *The usual power is 1000/1000 for OF and RF, and 998/1000 for RC. [#]The number of districts in the true cluster for model F is 8.

Table A7. Estimated bivariate power distributions P(l,s) × 1,000 of the 7 methods for cluster model G (RR = 1.3).

	CS									ES							
	Included *s* hot-spot districts									Included *s* hot-spot districts							
l	2	3	4	5	6	7	$8^{\#}$	Total	*l*	2	3	4	5	6	7	$8^{\#}$	Total
1-5	0	0	0	52	0	0	0	52	1-5	0	1	0	3	0	0	0	4
6	0	0	0	0	213	0	0	213	6	0	0	0	0	54	0	0	54
7	0	0	0	0	0	412	0	412	7	0	0	0	0	1	189	0	190
$8^{\#}$	0	0	0	1	0	22	0	23	$8^{\#}$	0	0	0	0	0	2	670	672
9	0	0	0	0	0	12	266	278	9	0	0	0	0	0	1	66	67
10	0	0	0	0	0	1	12	13	10	0	0	0	0	0	1	8	9
11	0	0	0	0	0	0	3	3	11	0	0	0	0	0	0	1	1
12	0	0	0	0	0	0	3	3	12	0	0	0	0	0	0	2	2
13	0	0	0	0	0	0	1	1	13	0	0	0	0	0	0	0	0
14	0	0	0	0	0	0	0	0	14	0	0	0	0	0	0	0	0
15	0	0	0	0	0	0	0	0	15	0	0	0	0	0	0	0	0
16	0	0	0	0	0	0	0	0	16	0	0	0	0	0	0	0	0
17-23	0	0	0	0	0	0	2	2	17-33	0	0	0	0	0	0	1	1
Total*	0	0	0	53	213	447	287	1000	Total*	0	1	0	3	55	193	748	1000

	GCS									GES							
	Included *s* hot-spot districts									Included *s* hot-spot districts							
l	2	3	4	5	6	7	$8^{\#}$	Total	*l*	2	3	4	5	6	7	$8^{\#}$	Total
1-5	0	0	0	43	0	0	0	43	1-5	0	1	0	2	0	0	0	3
6	0	0	0	9	173	0	0	182	6	0	0	0	0	51	0	0	51
7	0	0	0	1	24	364	0	389	7	0	0	0	0	4	183	0	187
$8^{\#}$	0	0	0	1	4	71	5	81	8*	0	0	0	0	1	14	619	634
9	0	0	0	1	0	12	264	277	9	0	0	0	0	0	3	95	98
10	0	0	0	0	0	2	16	18	10	0	0	0	0	0	3	17	20
11	0	0	0	0	0	0	4	4	11	0	0	0	0	0	3	1	4
12	0	0	0	0	0	0	3	3	12	0	0	0	0	0	0	2	2
13	0	0	0	0	0	0	1	1	13	0	0	0	0	0	0	0	0
14	0	0	0	0	0	0	0	0	14	0	0	0	0	0	0	0	0
15	0	0	0	0	0	0	0	0	15	0	0	0	0	0	0	0	0
16-23	0	0	0	0	0	0	2	2	16-33	0	0	0	0	0	0	1	1
Total*	0	0	0	55	201	449	295	1000	Total*	0	1	0	2	56	206	735	1000

CS: Circular spatial scan statistic, ES: Elliptic spatial scan statistic, GCS: Circular spatial scan statistic using Gini coefficient, GES: Elliptic spatial scan statistic using Gini coefficient, OF: Flexible spatial scan statistic, RC: Circular spatial scan statistic with a restricted likelihood ratio. RF: Flexible spatial scan statistic with a restricted likelihood ratio. 1000 trials were carried out. *The usual power is 1000/1000. #The number of districts in the true cluster for model G is 8.

Table A7. Estimated bivariate power distributions P(l,s) × 1,000 of the 7 methods for cluster model G (RR = 1.3) (continued).

	OF									RC							
	Included *s* hot-spot districts									Included *s* hot-spot districts							
l	2	3	4	5	6	7	8*	Total	*l*	2	3	4	5	6	7	8*	Total
1-6	0	0	1	3	52	0	0	56	1-6	0	3	15	89	291	0	0	398
7	0	0	0	0	3	213	0	216	7	0	0	0	1	4	384	0	389
8*	0	0	0	0	2	71	444	517	8*	0	0	0	0	1	18	142	161
9	0	0	0	0	2	16	139	157	9	0	0	0	0	0	2	42	44
10	0	0	0	0	2	3	36	41	10	0	0	0	0	0	0	6	6
11	0	0	0	0	0	1	8	9	11	0	0	0	0	0	0	1	1
12	0	0	0	0	0	0	4	4	12	0	0	0	0	0	0	1	1
13	0	0	0	0	0	0	0	0	13	0	0	0	0	0	0	0	0
Total*	0	0	1	3	61	304	631	1000	Total*	0	3	15	90	296	404	192	1000

	RF							
	Included *s* hot-spot districts							
l	2	3	4	5	6	7	8*	Total
1-6	0	0	2	7	55	0	0	64
7	0	0	0	0	6	217	0	223
8*	0	0	0	0	2	73	445	520
9	0	0	0	0	2	14	136	152
10	0	0	0	0	0	3	30	33
11	0	0	0	0	0	1	5	6
12-13	0	0	0	0	0	0	2	2
Total*	0	0	2	7	65	308	618	1000

CS: Circular spatial scan statistic, ES: Elliptic spatial scan statistic, GCS: Circular spatial scan statistic using Gini coefficient, GES: Elliptic spatial scan statistic using Gini coefficient, OF: Flexible spatial scan statistic, RC: Circular spatial scan statistic with a restricted likelihood ratio. RF: Flexible spatial scan statistic with a restricted likelihood ratio. 1000 trials were carried out. *The usual power is 1000/1000. #The number of districts in the true cluster for model G is 8.

Table B1. Estimated bivariate power distributions P(l,s) × 1,000 of the 7 methods for cluster model B (RR = 1.5).

	CS									ES							
	Included *s* hot-spot districts									Included *s* hot-spot districts							
l	5-6	7	8	9	10	11	$12^{\#}$	Total	*l*	5-6	7	8	9	10	11	$12^{\#}$	Total
5	0	0	0	0	0	0	0		5	4	0	0	0	0	0	0	4
6	1	0	0	0	0	0	0	1	6	35	0	0	0	0	0	0	35
7	26	4	0	0	0	0	0	30	7	0	293	0	0	0	0	0	293
8	0	18	4	0	0	0	0	22	8	0	164	1	0	0	0	0	165
9	3	3	26	1	0	0	0	33	9	1	31	19	7	0	0	0	58
10	0	6	25	9	0	0	0	40	10	0	0	65	29	1	0	0	95
11	0	20	7	29	4	0	0	60	11	0	0	33	2	5	0	0	40
$12^{\#}$	0	51	281	15	56	0	0	403	$12^{\#}$	0	0	27	9	22	7	0	65
13	0	0	35	205	16	28	0	284	13	0	0	0	2	38	0	0	40
14	0	0	13	5	60	8	1	87	14	0	0	0	27	4	0	0	31
15	0	0	1	7	1	3	0	12	15	0	1	0	4	1	5	0	11
16	0	0	1	0	3	1	0	5	16	0	0	1	6	0	23	0	30
17	0	0	1	0	1	1	0	3	17	0	0	0	4	4	0	0	8
18	0	0	0	1	4	0	0	5	18	0	0	0	25	19	0	0	44
19	0	0	0	0	0	2	0	2	19	0	0	0	26	29	0	2	57
20	0	0	0	0	0	0	0	0	20	0	0	0	0	1	0	0	1
21	0	0	0	0	0	1	0	1	21	0	0	0	0	1	0	1	2
22	0	0	0	0	0	0	0	0	22	0	0	0	0	0	3	0	3
23	0	0	0	0	0	0	0	0	23	0	0	0	0	0	1	16	17
24	0	0	0	0	0	0	0	0	24	0	0	0	0	0	0	0	0
25	0	0	0	0	0	0	0	0	25	0	0	0	0	0	1	0	1
26	0	0	0	7	0	0	0	7	26	0	0	0	0	0	0	0	0
27	0	0	0	0	5	0	0	5	27	0	0	0	0	0	0	0	0
Total*	30	102	394	279	150	44	1	1000	Total*	40	489	146	141	125	40	19	1000

	GCS									GES							
	Included *s* hot-spot districts									Included *s* hot-spot districts							
l	6	7	8	9	10	11	$12^{\#}$	Total	*l*	6	7	8	9	10	11	$12^{\#}$	Total
8	0	0	5	0	0	0	0	5	8	0	0	1	0	0	0	0	1
9	0	0	1	28	0	0	0	29	9	0	0	0	9	0	0	0	9
10	0	0	0	5	166	0	0	171	10	0	0	0	8	65	0	0	73
11	0	0	0	4	15	449	0	468	11	0	0	0	1	37	121	0	159
$12^{\#}$	0	0	0	0	22	29	199	250	$12^{\#}$	0	0	0	0	25	243	2	270
13	0	0	0	0	0	39	13	52	13	0	0	0	1	8	142	32	183
14	0	0	0	0	2	2	11	15	14	0	0	0	0	3	45	149	197
15	0	0	0	0	0	1	0	1	15	0	0	0	0	0	8	60	68
16	0	0	0	0	0	4	0	4	16	0	0	0	0	0	6	14	20
17	0	0	0	0	0	0	2	2	17	0	0	0	0	0	0	11	11
18	0	0	0	0	0	0	0	0	18	0	0	0	0	4	0	0	4
19	0	0	0	0	0	1	1	2	19	0	0	0	0	0	0	3	3
20	0	0	0	0	0	0	0	0	20	0	0	0	0	1	0	0	1
21	0	0	0	0	0	1	0	1	21	0	0	0	0	0	0	0	0
22	0	0	0	0	0	0	0	0	22	0	0	0	0	0	0	0	0
23	0	0	0	0	0	0	0	0	23	0	0	0	0	0	0	1	1
Total*	0	0	6	37	205	526	226	1000	Total*	0	0	1	19	143	565	272	1000

CS: Circular spatial scan statistic, ES: Elliptic spatial scan statistic, GCS: Circular spatial scan statistic using Gini coefficient, GES: Elliptic spatial scan statistic using Gini coefficient, OF: Flexible spatial scan statistic, RC: Circular spatial scan statistic with a restricted likelihood ratio. RF: Flexible spatial scan statistic with a restricted likelihood ratio. 1000 trials were carried out. *The usual power is 1000/1000. #The number of districts in the true cluster for model B is 12.

Table B1. Estimated bivariate power distributions P(l,s) × 1,000 of the 7 methods for cluster model B (RR = 1.5) (continued).

	OF									RC							
	Included *s* hot-spot districts									Included *s* hot-spot districts							
l	6	7	8	9	10	11	12#	Total	*l*	6	7	8	9	10	11	12#	Total
8	0	0	0	0	0	0	0	0	8	0	0	2	0	0	0	0	2
9	0	0	0	17	0	0	0	17	9	0	0	0	16	0	0	0	16
10	0	0	0	1	74	0	0	75	10	0	0	0	0	73	0	0	73
11	0	0	0	0	9	57	0	66	11	0	0	0	0	6	481	0	487
12#	0	0	0	0	0	4	751	755	12#	0	0	0	0	0	41	346	387
13	0	0	0	0	0	0	80	80	13	0	0	0	0	0	1	33	34
14	0	0	0	0	0	0	6	6	14	0	0	0	0	0	0	0	0
15	0	0	0	0	0	0	1	1	15	0	0	0	0	0	0	1	1
Total*	0	0	0	18	83	61	838	1000	Total*	0	0	2	16	79	523	380	1000

	RF							
	Included *s* hot-spot districts							
l	6	7	8	9	10	11	12#	Total
8	0	0	0	11	0	0	0	11
9	0	0	0	1	51	0	0	52
10	0	0	0	0	4	61	0	65
11	0	0	0	0	0	4	778	782
12#	0	0	0	0	0	0	84	84
13	0	0	0	0	0	0	6	6
14	0	0	0	0	0	0	0	0
Total*	0	0	0	12	55	65	868	1000

CS: Circular spatial scan statistic, ES: Elliptic spatial scan statistic, GCS: Circular spatial scan statistic using Gini coefficient, GES: Elliptic spatial scan statistic using Gini coefficient, OF: Flexible spatial scan statistic, RC: Circular spatial scan statistic with a restricted likelihood ratio. RF: Flexible spatial scan statistic with a restricted likelihood ratio. 1000 trials were carried out. *The usual power is 1000/1000. #The number of districts in the true cluster for model B is 12.

Table B2. Estimated bivariate power distributions P(l,s) × 1,000 of the 7 methods for cluster model C (RR = 1.5).

	CS									ES							
	Included *s* hot-spot districts									Included *s* hot-spot districts							
l	5-6	7-8	9-10	11	12	13	$14^{\#}$	Total	*l*	8	9	10	11	12	13	$14^{\#}$	Total
5	17	0	0	0	0	0	0	17	5	0	0	0	0	0	0	0	0
6	42	0	0	0	0	0	0	42	6	0	0	0	0	0	0	0	0
7	32	37	0	0	0	0	0	69	7	0	0	0	0	0	0	0	0
8	0	87	0	0	0	0	0	87	8	1	0	0	0	0	0	0	1
9	28	52	3	0	0	0	0	83	9	0	174	0	0	0	0	0	174
10	0	73	6	0	0	0	0	79	10	0	2	1	0	0	0	0	3
11	0	35	51	0	0	0	0	87	11	0	0	89	17	0	0	0	106
12	0	13	80	0	0	0	0	93	12	0	0	3	53	208	0	0	264
13	0	1	127	29	0	0	0	157	13	0	0	0	11	231	0	0	242
$14^{\#}$	0	1	13	21	8	0	0	43	$14^{\#}$	0	0	0	5	185	0	0	190
15	0	0	75	63	4	0	0	142	15	0	0	0	0	10	3	0	13
16	0	0	2	14	5	0	0	21	16	0	0	0	0	4	1	0	5
17	0	0	1	2	14	5	0	22	17	0	0	0	0	1	0	0	1
18	0	0	1	1	1	49	0	52	18	0	0	0	0	1	0	0	1
19	0	0	0	0	0	3	0	3	19	0	0	0	0	0	0	0	0
20	0	0	0	0	0	2	0	2	20	0	0	0	0	0	0	0	0
21-26	0	0	0	0	0	1	0	1	21	0	0	0	0	0	0	0	0
Total*	119	300	359	130	32	60	0	1000	Total*	1	176	93	86	640	4	0	1000

	GCS									GES							
	Included *s* hot-spot districts									Included *s* hot-spot districts							
l	7-8	9	10	11	12	13	14*	Total	*l*	8	9	10	11	12	13	$14^{\#}$	Total
7	1	0	0	0	0	0	0	1	7	0	0	0	0	0	0	0	0
8	1	0	0	0	0	0	0	1	8	0	0	0	0	0	0	0	0
9	0	17	0	0	0	0	0	17	9	0	1	0	0	0	0	0	1
10	0	1	80	0	0	0	0	81	10	0	0	5	0	0	0	0	5
11	0	3	15	134	0	0	0	152	11	0	0	2	30	0	0	0	32
12	0	1	17	57	88	0	0	163	12	0	0	0	18	283	0	0	301
13	0	0	13	34	84	29	0	160	13	0	0	0	6	209	18	0	233
$14^{\#}$	0	0	3	13	63	76	0	155	$14^{\#}$	0	0	0	3	103	80	0	186
15	0	0	3	22	22	56	22	125	15	0	0	1	4	20	68	29	122
16	0	0	0	2	16	17	15	50	16	0	0	0	2	4	36	28	70
17	0	0	0	1	9	25	7	42	17	0	0	0	0	2	18	9	29
18	0	0	1	0	3	42	2	48	18	0	0	0	0	1	11	1	13
19	0	0	0	0	0	2	1	3	19	0	0	0	0	0	4	2	6
20	0	0	0	0	0	2	0	2	20-21	0	0	0	0	0	1	1	2
Total*	2	22	132	263	285	249	47	1000	Total*	0	1	8	63	622	236	70	1000

CS: Circular spatial scan statistic, ES: Elliptic spatial scan statistic, GCS: Circular spatial scan statistic using Gini coefficient, GES: Elliptic spatial scan statistic using Gini coefficient, OF: Flexible spatial scan statistic, RC: Circular spatial scan statistic with a restricted likelihood ratio. RF: Flexible spatial scan statistic with a restricted likelihood ratio. 1000 trials were carried out. *The usual power is 1000/1000. #The number of districts in the true cluster for model C is 14.

Table B2. Estimated bivariate power distributions P(l,s) × 1,000 of the 7 methods for cluster model C (RR = 1.5) (continued).

	OF									RC							
	Included *s* hot-spot districts									Included *s* hot-spot districts							
l	8	9	10	11	12	13	14#	Total	*l*	6-7	8-9	10	11	12	13	14#	Total
6	0	0	0	0	0	0	0	0	6	2	0	0	0	0	0	0	2
7	0	0	0	0	0	0	0	0	7	0	0	0	0	0	0	0	0
8	0	0	0	0	0	0	0	0	8	1	17	0	0	0	0	0	18
9	0	0	0	0	0	0	0	0	9	0	93	0	0	0	0	0	93
10	0	1	14	0	0	0	0	15	10	0	11	198	0	0	0	0	209
11	0	0	3	92	0	0	0	95	11	0	0	24	273	0	0	0	297
12	0	0	0	20	85	0	0	105	12	0	0	3	41	170	0	0	214
13	0	0	0	4	45	57	0	106	13	0	0	0	6	60	56	0	122
14#	0	0	0	0	6	145	0	151	14#	0	0	0	0	3	26	8	37
15	0	0	0	0	1	10	463	474	15	0	0	0	0	0	3	5	8
16	0	0	0	0	0	1	49	50	16	0	0	0	0	0	0	0	0
17	0	0	0	0	0	0	4	4	17	0	0	0	0	0	0	0	0
Total*	0	1	17	116	137	213	516	1000		3	121	225	320	233	85	13	1000

	RF							
	Included *s* hot-spot districts							
l	8	9	10	11	12	13	14*	Total
10	0	0	18	0	0	0	0	18
11	0	0	3	110	0	0	0	113
12	0	0	0	17	199	0	0	216
13	0	0	0	1	44	264	0	309
14#	0	0	0	0	5	70	174	249
15	0	0	0	0	0	7	78	85
16	0	0	0	0	0	1	9	10
Total*	0	0	21	128	248	342	261	1000

CS: Circular spatial scan statistic, ES: Elliptic spatial scan statistic, GCS: Circular spatial scan statistic using Gini coefficient, GES: Elliptic spatial scan statistic using Gini coefficient, OF: Flexible spatial scan statistic, RC: Circular spatial scan statistic with a restricted likelihood ratio. RF: Flexible spatial scan statistic with a restricted likelihood ratio. 1000 trials were carried out. *The usual power is 1000/1000. #The number of districts in the true cluster for model C is 14.

Table B3. Estimated bivariate power distributions P(l,s) × 1,000 of the 7 methods for cluster model D (RR = 1.5).

	CS									ES							
	Included *s* hot-spot districts									Included *s* hot-spot districts							
l	1	2	3	4	5	6	7[#]	Total	*l*	1	2	3	4	5	6	7[#]	Total
2	0	1	0	0	0	0	0	1	3	0	0	49	0	0	0	0	49
3	0	0	33	0	0	0	0	33	4	0	0	12	671	0	0	0	683
4	0	0	1	3	0	0	0	4	5	0	0	1	0	3	0	0	4
5	0	0	0	87	0	0	0	87	6	0	0	0	111	10	5	0	126
6	0	0	0	7	13	0	0	20	7[#]	0	0	0	57	13	2	0	72
7[#]	0	0	0	16	2	1	0	19	8	0	0	0	0	2	1	0	3
8	0	0	0	1	159	0	0	160	9	0	0	0	0	12	5	0	17
9	0	0	0	0	3	23	0	26	10	0	0	0	0	8	13	0	21
10	0	0	0	0	0	572	0	572	11	0	0	0	0	1	1	0	2
11	0	0	0	0	0	9	9	18	12	0	0	0	0	5	1	2	8
12	0	0	0	0	0	5	0	5	13	0	0	0	0	1	3	2	6
13	0	0	0	0	0	0	2	2	14	0	0	0	0	0	5	0	5
14	0	0	0	0	0	2	14	16	15	0	0	0	0	0	2	0	2
15	0	0	0	0	0	4	1	5	16	0	0	0	0	0	0	2	2
16-26	0	0	0	0	0	9	23	32	17	0	0	0	0	0	0	0	0
Total*	0	0	34	114	177	625	49	1000	Total*	0	0	62	839	55	38	6	1000

	GCS									GES							
	Included *s* hot-spot districts									Included *s* hot-spot districts							
l	1	2	3	4	5	6	7[#]	Total	*l*	1	2	3	4	5	6	7[#]	Total
4	0	0	0	7	0	0	0	7	4	0	0	0	1	0	0	0	1
5	0	0	0	0	75	0	0	75	5	0	0	0	0	5	0	0	5
6	0	0	0	1	17	242	0	260	6	0	0	0	0	3	36	0	39
7[#]	0	0	0	1	24	133	128	286	7[#]	0	0	0	0	3	24	506	533
8	0	0	0	0	23	96	39	158	8	0	0	0	0	1	22	142	165
9	0	0	0	0	2	34	18	54	9	0	0	0	0	0	10	148	158
10	0	0	0	0	0	78	30	108	10	0	0	0	0	1	11	44	56
11	0	0	0	0	0	6	23	29	11	0	0	0	0	0	12	8	20
12	0	0	0	0	0	1	6	7	12	0	0	0	0	0	2	7	9
13	0	0	0	0	0	0	2	2	13	0	0	0	0	1	3	2	6
14	0	0	0	0	0	1	6	7	14	0	0	0	0	0	3	4	7
15	0	0	0	0	0	2	0	2	15	0	0	0	0	0	0	0	0
16-22	0	0	0	0	0	3	2	5	16	0	0	0	0	0	0	1	1
Total*	0	0	0	9	141	596	254	1000	Total*	0	0	0	1	14	123	862	1000

CS: Circular spatial scan statistic, ES: Elliptic spatial scan statistic, GCS: Circular spatial scan statistic using Gini coefficient, GES: Elliptic spatial scan statistic using Gini coefficient, OF: Flexible spatial scan statistic, RC: Circular spatial scan statistic with a restricted likelihood ratio. RF: Flexible spatial scan statistic with a restricted likelihood ratio. 1000 trials were carried out. *The usual power is 1000/1000. [#]The number of districts in the true cluster for model D is 7.

Table B3. Estimated bivariate power distributions P(l,s) × 1,000 of the 7 methods for cluster model D (RR = 1.5) (continued).

	OF									RC							
	Included *s* hot-spot districts									Included *s* hot-spot districts							
l	1	2	3	4	5	6	7#	Total	*l*	1	2	3	4	5	6	7#	Total
4	0	0	0	2	0	0	0	2	4	0	0	0	9	0	0	0	9
5	0	0	0	1	5	0	0	6	5	0	0	0	0	123	0	0	123
6	0	0	0	2	7	426	0	435	6	0	0	0	0	9	457	0	466
7#	0	0	0	1	0	63	12	76	7#	0	0	0	0	0	54	296	350
8	0	0	0	0	0	14	401	415	8	0	0	0	0	0	5	43	48
9	0	0	0	0	0	0	49	49	9	0	0	0	0	0	0	3	3
10	0	0	0	0	0	0	9	9	10	0	0	0	0	0	0	1	1
11	0	0	0	0	0	0	3	3	11	0	0	0	0	0	0	1	1
12	0	0	0	0	0	0	2	2	12	0	0	0	0	0	0	0	0
13	0	0	0	0	0	1	2	3	13	0	0	0	0	0	0	0	0
Total*	0	0	0	6	12	504	478	1000	Total*	0	0	0	9	132	516	343	1000

	RF							
	Included *s* hot-spot districts							
l	1	2	3	4	5	6	7#	Total
4	0	0	0	2	0	0	0	2
5	0	0	0	0	7	0	0	7
6	0	0	0	0	0	617	0	617
7#	0	0	0	0	0	40	191	231
8	0	0	0	0	0	10	117	127
9	0	0	0	0	0	0	16	16
Total*	0	0	0	2	7	667	324	1000

CS: Circular spatial scan statistic, ES: Elliptic spatial scan statistic, GCS: Circular spatial scan statistic using Gini coefficient, GES: Elliptic spatial scan statistic using Gini coefficient, OF: Flexible spatial scan statistic, RC: Circular spatial scan statistic with a restricted likelihood ratio. RF: Flexible spatial scan statistic with a restricted likelihood ratio. 1000 trials were carried out. *The usual power is 1000/1000. #The number of districts in the true cluster for model D is 7.

Table B4. Estimated bivariate power distributions P(l,s) × 1,000 of the 7 methods for cluster model E (RR = 1.5).

CS

l	Included *s* hot-spot districts: 10-12	13	14	15	16	17	18[#]	Total
11	1	0	0	0	0	0	0	1
12	4	0	0	0	0	0	0	4
13	4	14	0	0	0	0	0	18
14	2	8	7	0	0	0	0	17
15	0	1	15	16	0	0	0	32
16	0	0	31	51	16	0	0	98
17	0	2	11	5	240	30	0	288
18[#]	0	2	2	23	62	10	0	99
19	0	0	3	7	39	155	26	230
20	0	0	0	1	2	44	1	48
21	1	0	0	2	5	17	14	39
22	0	0	6	1	0	9	22	38
23	0	0	0	0	0	0	2	2
24	0	0	0	4	9	1	3	17
25	0	0	0	0	1	56	1	58
26	0	0	0	0	0	5	2	7
27	0	0	0	0	0	1	0	1
28	0	0	0	0	0	0	0	0
29	0	0	0	0	0	2	1	3
Total*	12	27	75	110	374	330	72	1000

ES

l	Included *s* hot-spot districts: 10-12	13	14	15	16	17	18[#]	Total
10	10	0	0	0	0	0	0	10
11	52	0	0	0	0	0	0	52
12	16	18	0	0	0	0	0	34
13	0	45	2	0	0	0	0	47
14	0	11	36	7	0	0	0	54
15	0	0	134	6	40	0	0	180
16	0	0	18	84	2	22	0	126
17	0	0	5	207	8	3	2	225
18[#]	0	0	1	10	152	1	10	174
19	0	0	0	2	8	12	0	22
20	0	0	0	1	14	51	0	66
21	0	0	0	0	1	4	0	5
22	0	0	0	0	1	0	0	1
23	0	0	0	0	0	0	4	4
24	0	0	0	0	0	0	0	0
25	0	0	0	0	0	0	0	0
26	0	0	0	0	0	0	0	0
27	0	0	0	0	0	0	0	0
28	0	0	0	0	0	0	0	0
Total*	78	74	196	317	226	93	16	1000

GCS

l	Included *s* hot-spot districts: 12	13	14	15	16	17	18[#]	Total
13	0	3	0	0	0	0	0	3
14	0	1	15	0	0	0	0	16
15	0	0	2	35	0	0	0	37
16	0	0	0	5	92	0	0	97
17	0	0	1	1	16	487	0	505
18[#]	0	0	0	0	4	13	226	243
19	0	0	1	3	2	21	20	47
20	0	0	0	0	0	17	3	20
21	0	0	0	0	0	2	12	14
22	0	0	0	0	0	2	10	12
23	0	0	0	0	0	0	1	1
24	0	0	0	0	0	0	1	1
25	0	0	0	0	0	1	1	2
26	0	0	0	0	0	0	1	1
27	0	0	0	0	0	1	0	1
Total*	0	4	19	44	114	544	275	1000

GES

l	Included *s* hot-spot districts: 12	13	14	15	16	17	18[#]	Total
13	0	1	0	0	0	0	0	1
14	0	0	46	0	0	0	0	46
15	0	1	9	178	0	0	0	188
16	0	1	5	62	275	0	0	343
17	0	0	2	7	78	120	0	207
18[#]	0	0	1	11	19	82	37	150
19	0	0	0	0	3	41	3	47
20	0	0	0	0	4	5	2	11
21	0	0	0	0	1	1	2	4
22	0	0	0	0	0	1	0	1
23	0	0	0	0	0	2	0	2
24	0	0	0	0	0	0	0	0
25	0	0	0	0	0	0	0	0
26	0	0	0	0	0	0	0	0
27	0	0	0	0	0	0	0	0
Total*	0	3	63	258	380	252	44	1000

CS: Circular spatial scan statistic, ES: Elliptic spatial scan statistic, GCS: Circular spatial scan statistic using Gini coefficient, GES: Elliptic spatial scan statistic using Gini coefficient, OF: Flexible spatial scan statistic, RC: Circular spatial scan statistic with a restricted likelihood ratio. RF: Flexible spatial scan statistic with a restricted likelihood ratio. 1000 trials were carried out. *The usual power is 1000/1000. [#]The number of districts in the true cluster for model E is 18.

Table B4. Estimated bivariate power distributions P(l,s) × 1,000 of the 7 methods for cluster model E (RR = 1.5) (continued).

	OF								RC								
	Included *s* hot-spot districts									Included *s* hot-spot districts							
l	12	13	14	15	16	17	18#	Total	*l*	12	13	14	15	16	17	18#	Total
13	0	0	0	0	0	0	0	0	13	0	4	0	0	0	0	0	4
14	1	4	1	0	0	0	0	6	14	0	0	16	0	0	0	0	16
15	0	6	8	1	0	0	0	15	15	0	0	3	59	0	0	0	62
16	0	1	14	66	10	0	0	91	16	0	0	0	4	131	0	0	135
17	0	0	6	53	128	0	0	187	17	0	0	0	0	11	440	0	451
18#	0	0	0	7	220	39	0	266	18#	0	0	0	0	0	24	286	310
19	0	0	0	1	19	339	11	370	19	0	0	0	0	0	0	22	22
20	0	0	0	0	1	17	40	58	20	0	0	0	0	0	0	0	0
21	0	0	0	0	1	2	3	6	21	0	0	0	0	0	0	0	0
22	0	0	0	0	0	0	1	1	22	0	0	0	0	0	0	0	0
Total*	1	11	29	128	379	397	55	1000	Total*	0	4	19	63	142	464	308	1000

	RF							
	Included *s* hot-spot districts							
l	12	13	14	15	16	17	18#	Total
13	0	1	0	0	0	0	0	1
14	0	0	11	0	0	0	0	11
15	0	0	3	72	0	0	0	75
16	0	0	0	10	325	0	0	335
17	0	0	0	0	42	148	0	190
18#	0	0	0	0	3	29	330	362
19	0	0	0	0	0	25	25	0
20	0	0	0	0	0	1	1	0
Total*	0	1	14	82	370	177	356	1000

CS: Circular spatial scan statistic, ES: Elliptic spatial scan statistic, GCS: Circular spatial scan statistic using Gini coefficient, GES: Elliptic spatial scan statistic using Gini coefficient, OF: Flexible spatial scan statistic, RC: Circular spatial scan statistic with a restricted likelihood ratio. RF: Flexible spatial scan statistic with a restricted likelihood ratio. 1000 trials were carried out. *The usual power is 1000/1000. #The number of districts in the true cluster for model E is 18.

Table B5. Estimated bivariate power distributions P(l,s) × 1,000 of the 7 methods for cluster model F (RR = 1.5).

	CS									ES							
	Included *s* hot-spot districts									Included *s* hot-spot districts							
l	2	3	4	5	6	7	8[#]	Total	*l*	2	3	4	5	6	7	8[#]	Total
4	0	0	1	0	0	0	0	1	4	0	0	0	0	0	0	0	0
5	0	0	0	4	0	0	0	4	5	0	0	0	7	0	0	0	7
6	0	0	0	3	7	0	0	10	6	0	0	0	0	23	0	0	23
7	0	0	0	0	127	0	0	127	7	0	0	0	0	239	21	0	260
8[#]	0	0	0	0	14	167	0	181	8[#]	0	0	0	0	0	569	51	620
9	0	0	0	2	2	178	5	187	9	0	0	0	0	0	9	8	17
10	0	0	0	0	42	67	180	289	10	0	0	0	0	0	19	0	19
11	0	0	0	0	0	51	57	108	11	0	0	0	0	0	0	49	49
12	0	0	0	0	3	4	16	23	12	0	0	0	0	0	1	2	3
13	0	0	0	0	13	4	8	25	13	0	0	0	0	0	0	1	1
14	0	0	0	0	1	21	0	22	14	0	0	0	0	0	0	1	1
15	0	0	0	0	0	2	12	14	15	0	0	0	0	0	0	0	0
16	0	0	0	0	0	0	1	1	16	0	0	0	0	0	0	0	0
17	0	0	0	0	0	0	1	1	17	0	0	0	0	0	0	0	0
18	0	0	0	0	1	2	0	3	18	0	0	0	0	0	0	0	0
19	0	0	0	0	0	0	1	1	19	0	0	0	0	0	0	0	0
20	0	0	0	0	0	1	0	1	20	0	0	0	0	0	0	0	0
21-29	0	0	0	0	0	1	1	2	21	0	0	0	0	0	0	0	0
Total*	0	0	1	9	210	498	282	1000	Total*	0	0	0	7	262	619	112	1000

	GCS									GES							
	Included *s* hot-spot districts									Included *s* hot-spot districts							
l	2	3	4	5	6	7	8[#]	Total	*l*	12	13	14	15	16	17	18[#]	Total
4	0	0	0	0	0	0	0	0	4	0	0	0	0	0	0	0	0
5	0	0	0	4	0	0	0	4	5	0	0	0	0	0	0	0	0
6	0	0	0	0	25	0	0	25	6	0	0	0	0	16	0	0	16
7	0	0	0	0	10	196	0	206	7	0	0	0	0	4	203	0	207
8[#]	0	0	0	0	4	48	299	351	8[#]	0	0	0	0	1	48	356	405
9	0	0	0	0	0	52	137	189	9	0	0	0	0	0	27	213	240
10	0	0	0	0	0	25	114	139	10	0	0	0	0	0	15	51	66
11	0	0	0	0	0	5	45	50	11	0	0	0	0	0	7	26	33
12	0	0	0	0	1	4	10	15	12	0	0	0	0	0	3	19	22
13	0	0	0	0	2	2	4	8	13	0	0	0	0	0	1	5	6
14	0	0	0	0	0	5	1	6	14	0	0	0	0	0	0	3	3
15	0	0	0	0	0	0	3	3	15	0	0	0	0	0	0	1	1
16	0	0	0	0	0	0	1	1	16	0	0	0	0	0	0	1	1
17	0	0	0	0	0	0	0	0	17	0	0	0	0	0	0	0	0
18	0	0	0	0	1	2	0	3	18	0	0	0	0	0	0	0	0
Total*	0	0	0	4	43	339	614	1000	Total*	0	0	0	0	21	304	675	1000

CS: Circular spatial scan statistic, ES: Elliptic spatial scan statistic, GCS: Circular spatial scan statistic using Gini coefficient, GES: Elliptic spatial scan statistic using Gini coefficient, OF: Flexible spatial scan statistic, RC: Circular spatial scan statistic with a restricted likelihood ratio. RF: Flexible spatial scan statistic with a restricted likelihood ratio. 1000 trials were carried out. *The usual power is 1000/1000. [#]The number of districts in the true cluster for model F is 8.

Table B5. Estimated bivariate power distributions P(l,s) × 1,000 of the 7 methods for cluster model F (RR = 1.5) (continued).

OF								
	Included *s* hot-spot districts							
l	2	3	4	5	6	7	8#	Total
6	0	0	0	0	0	0	0	0
7	0	0	0	0	0	0	0	0
8#	0	0	0	0	0	4	1	5
9	0	0	0	0	0	6	170	176
10	0	0	0	0	0	5	634	639
11	0	0	0	0	0	0	159	159
12	0	0	0	0	0	0	18	18
13	0	0	0	0	0	0	3	3
Total*	0	0	0	0	0	15	985	1000

RC								
	Included *s* hot-spot districts							
l	2	3	4	5	6	7	8#	Total
6	0	0	0	0	22	0	0	22
7	0	0	0	0	1	209	0	210
8#	0	0	0	0	0	42	570	612
9	0	0	0	0	0	1	143	144
10	0	0	0	0	0	0	10	10
11	0	0	0	0	0	0	2	2
12	0	0	0	0	0	0	0	0
13	0	0	0	0	0	0	0	0
Total*	0	0	0	0	23	252	725	1000

RF								
	Included *s* hot-spot districts							
l	2	3	4	5	6	7	8#	Total
6	0	0	0	0	0	0	0	0
7	0	0	0	0	0	29	0	29
8#	0	0	0	0	0	6	725	731
9	0	0	0	0	0	2	194	196
10	0	0	0	0	0	1	41	42
11	0	0	0	0	0	0	1	1
12	0	0	0	0	0	0	1	1
Total*	0	0	0	0	0	38	962	1000

CS: Circular spatial scan statistic, ES: Elliptic spatial scan statistic, GCS: Circular spatial scan statistic using Gini coefficient, GES: Elliptic spatial scan statistic using Gini coefficient, OF: Flexible spatial scan statistic, RC: Circular spatial scan statistic with a restricted likelihood ratio. RF: Flexible spatial scan statistic with a restricted likelihood ratio. 1000 trials were carried out. *The usual power is 1000/1000. #The number of districts in the true cluster for model F is 8.

Table B6. Estimated bivariate power distributions P(l,s) × 1,000 of the 7 methods for cluster model G (RR = 1.5).

	CS									ES							
	Included *s* hot-spot districts									Included *s* hot-spot districts							
l	2	3	4	5	6	7	8#	Total	*l*	2	3	4	5	6	7	8#	Total
5	0	0	0	11	0	0	0	11	5	0	0	0	0	0	0	0	0
6	0	0	0	0	174	0	0	174	6	0	0	0	0	5	0	0	5
7	0	0	0	0	0	515	0	515	7	0	0	0	0	0	55	0	55
8#	0	0	0	0	0	1	0	1	8#	0	0	0	0	0	0	934	934
9	0	0	0	0	0	1	298	298	9	0	0	0	0	0	0	6	6
Total*	0	0	0	11	174	517	298	1000	Total*	0	0	0	0	5	55	940	1000

	GCS									GES							
	Included *s* hot-spot districts									Included *s* hot-spot districts							
l	2	3	4	5	6	7	8#	Total	*l*	2	3	4	5	6	7	8#	Total
6	0	0	0	0	50	0	0	50	6	0	0	0	0	1	0	0	1
7	0	0	0	0	7	278	0	284	7	0	0	0	0	0	39	0	39
8#	0	0	0	0	0	88	41	129	8#	0	0	0	0	0	12	757	769
9	0	0	0	0	0	9	482	491	9	0	0	0	0	1	19	89	109
10	0	0	0	0	0	0	31	31	10	0	0	0	0	1	12	44	57
11	0	0	0	0	0	0	12	12	11	0	0	0	0	0	21	2	23
12	0	0	0	0	0	0	2	2	12	0	0	0	0	0	0	2	2
Total*	0	0	0	0	57	375	568	1000	Total*	0	0	0	0	3	103	894	1000

CS: Circular spatial scan statistic, ES: Elliptic spatial scan statistic, GCS: Circular spatial scan statistic using Gini coefficient, GES: Elliptic spatial scan statistic using Gini coefficient, OF: Flexible spatial scan statistic, RC: Circular spatial scan statistic with a restricted likelihood ratio. RF: Flexible spatial scan statistic with a restricted likelihood ratio. 1000 trials were carried out. *The usual power is 1000/1000. #The number of districts in the true cluster for model G is 8.

Table B6. Estimated bivariate power distributions P(l,s) × 1,000 of the 7 methods for cluster model G (RR = 1.5) (continued).

	OF									RC							
	Included *s* hot-spot districts									Included *s* hot-spot districts							
l	2	3	4	5	6	7	8#	Total	*l*	2	3	4	5	6	7	8#	Total
6	0	0	0	0	2	0	0	2	6	0	0	0	0	34	0	0	34
7	0	0	0	0	0	88	0	88	7	0	0	0	0	0	214	0	214
8#	0	0	0	0	0	0	886	886	8#	0	0	0	0	0	1	720	721
9	0	0	0	0	0	0	24	24	9	0	0	0	0	0	1	30	31
10	0	0	0	0	0	0	0	0	10	0	0	0	0	0	0	0	0
11	0	0	0	0	0	0	0	0	11	0	0	0	0	0	0	0	0
12	0	0	0	0	0	0	0	0	12	0	0	0	0	0	0	0	0
13	0	0	0	0	0	0	0	0	13	0	0	0	0	0	0	0	0
Total*	0	0	0	0	2	88	910	1000	Total*	0	0	0	0	34	216	750	1000

	RF							
	Included *s* hot-spot districts							
l	2	3	4	5	6	7	8#	Total
6	0	0	0	0	2	0	0	2
7	0	0	0	0	0	88	0	88
8#	0	0	0	0	0	0	886	886
9	0	0	0	0	0	0	24	24
10	0	0	0	0	0	0	0	0
11	0	0	0	0	0	0	0	0
12	0	0	0	0	0	0	0	0
Total*	0	0	0	0	2	88	910	1000

CS: Circular spatial scan statistic, ES: Elliptic spatial scan statistic, GCS: Circular spatial scan statistic using Gini coefficient, GES: Elliptic spatial scan statistic using Gini coefficient, OF: Flexible spatial scan statistic, RC: Circular spatial scan statistic with a restricted likelihood ratio. RF: Flexible spatial scan statistic with a restricted likelihood ratio. 1000 trials were carried out. *The usual power is 1000/1000. #The number of districts in the true cluster for model G is 8.

Table C1. Estimated bivariate power distributions P(l,s) × 1,000 of the 7 methods for cluster model A (RR = 2).

CS	Included *s* hot-spot districts							
l	5	6	7	8	9	10	$11^{\#}$	Total
8	0	0	0	0	0	0	0	0
9	0	0	0	0	1	0	0	1
10	0	0	0	0	0	0	0	0
$11^{\#}$	0	0	0	0	1	0	0	1
12	0	0	0	0	0	0	0	0
13	0	0	0	0	0	757	0	757
14	0	0	0	0	0	0	227	227
15	0	0	0	0	0	0	0	0
16	0	0	0	0	0	0	0	0
17	0	0	0	0	0	0	14	0
18	0	0	0	0	0	0	0	0
Total*	0	0	0	0	2	757	241	1000

ES	Included *s* hot-spot districts							
l	5	6	7	8	9	10	$11^{\#}$	Total
8	0	0	0	664	0	0	0	664
9	0	0	0	2	2	0	0	4
10	0	0	0	0	26	10	0	36
$11^{\#}$	0	0	0	0	17	1	17	35
12	0	0	0	0	0	1	138	139
13	0	0	0	0	2	42	0	44
14	0	0	0	0	0	2	1	3
15	0	0	0	0	0	0	0	0
16	0	0	0	0	0	2	68	70
17	0	0	0	0	0	0	2	2
18	0	0	0	0	0	0	3	3
Total*	0	0	0	666	47	58	229	1000

GCS	Included *s* hot-spot districts							
l	5	6	7	8	9	10	$11^{\#}$	Total
9	0	0	0	0	0	0	0	0
10	0	0	0	0	0	451	0	451
$11^{\#}$	0	0	0	0	0	16	166	182
12	0	0	0	0	0	121	6	127
13	0	0	0	0	0	53	44	97
14	0	0	0	0	0	5	95	100
15	0	0	0	0	0	0	4	4
16	0	0	0	0	0	0	37	37
17	0	0	0	0	0	0	2	2
18	0	0	0	0	0	0	0	0
Total*	0	0	0	0	0	646	354	1000

GES	Included *s* hot-spot districts							
l	5	6	7	8	9	10	$11^{\#}$	Total
9	0	0	0	0	0	5	0	5
10	0	0	0	0	0	7	12	19
$11^{\#}$	0	0	0	0	0	3	200	203
12	0	0	0	0	0	1	607	608
13	0	0	0	0	0	0	85	85
14	0	0	0	0	0	0	47	47
15	0	0	0	0	0	0	30	30
16	0	0	0	0	0	0	2	2
17	0	0	0	0	0	0	1	1
18	0	0	0	0	0	5	0	5
Total*	0	0	0	0	0	16	984	1000

CS: Circular spatial scan statistic, ES: Elliptic spatial scan statistic, GCS: Circular spatial scan statistic using Gini coefficient, GES: Elliptic spatial scan statistic using Gini coefficient, OF: Flexible spatial scan statistic, RC: Circular spatial scan statistic with a restricted likelihood ratio. RF: Flexible spatial scan statistic with a restricted likelihood ratio. 1000 trials were carried out. *The usual power is 1000/1000. #The number of districts in the true cluster for model A is 11.

Table C1. Estimated bivariate power distributions P(l,s) × 1,000 of the 7 methods for cluster model A (RR = 2) (continued).

	OF									RC							
	Included *s* hot-spot districts									Included *s* hot-spot districts							
l	5	6	7	8	9	10	11#	Total	*l*	5	6	7	8	9	10	11#	Total
10	0	0	0	0	0	1	0	1	10	0	0	0	0	0	1	0	1
11#	0	0	0	0	0	3	166	169	11#	0	0	0	0	0	0	958	958
12	0	0	0	0	0	0	798	798	12	0	0	0	0	0	0	41	41
13	0	0	0	0	0	0	30	30	13	0	0	0	0	0	0	0	0
14	0	0	0	0	0	0	2	2	14	0	0	0	0	0	0	0	0
Total*	0	0	0	0	0	4	996	1000	Total*	0	0	0	0	0	1	999	1000

	RF							
	Included *s* hot-spot districts							
l	5	6	7	8	9	10	11#	Total
10	0	0	0	0	0	3	0	3
11#	0	0	0	0	0	0	800	800
12	0	0	0	0	0	0	192	192
13	0	0	0	0	0	0	4	4
Total*	0	0	0	0	0	3	997	1000

CS: Circular spatial scan statistic, ES: Elliptic spatial scan statistic, GCS: Circular spatial scan statistic using Gini coefficient, GES: Elliptic spatial scan statistic using Gini coefficient, OF: Flexible spatial scan statistic, RC: Circular spatial scan statistic with a restricted likelihood ratio. RF: Flexible spatial scan statistic with a restricted likelihood ratio. 1000 trials were carried out. *The usual power is 1000/1000. #The number of districts in the true cluster for model A is 11.

Table C2. Estimated bivariate power distributions P(l,s) × 1,000 of the 7 methods for cluster model B (RR = 2).

	CS									ES							
	Included *s* hot-spot districts									Included *s* hot-spot districts							
l	6	7	8	9	10	11	12[#]	Total	*l*	5-6	7	8	9	10	11	12[#]	Total
5	0	0	0	0	0	0	0	0	5	3	0	0	0	0	0	0	4
6	0	0	0	0	0	0	0	0	6	0	0	0	0	0	0	0	3
7	1	0	0	0	0	0	0	1	7	0	450	0	0	0	0	0	450
8	0	4	0	0	0	0	0	4	8	0	205	0	0	0	0	0	205
9	0	0	0	0	0	0	0	0	9	0	9	0	0	0	0	0	9
10	0	0	0	2	0	0	0	2	10	0	0	104	8	0	0	0	112
11	0	0	0	4	0	0	0	4	11	0	0	39	0	0	0	0	39
12[#]	0	4	152	1	2	0	0	159	12[#]	0	0	9	2	34	1	0	46
13	0	0	24	740	4	11	0	779	13	0	0	0	0	34	0	0	34
14	0	0	0	0	43	1	0	44	14	0	0	0	8	0	0	0	8
15	0	0	0	4	0	3	0	7	15	0	0	0	1	0	5	0	6
16	0	0	0	0	0	0	0	0	16	0	0	0	3	0	12	0	15
17	0	0	0	0	0	0	0	0	17	0	0	0	4	1	0	0	5
18	0	0	0	0	0	0	0	0	18	0	0	0	13	4	0	0	17
19	0	0	0	0	0	0	0	0	19	0	0	0	7	28	0	0	35
20	0	0	0	0	0	0	0	0	20	0	0	0	0	0	0	0	0
21	0	0	0	0	0	0	0	0	21	0	0	0	0	0	0	0	0
22	0	0	0	0	0	0	0	0	22	0	0	0	0	0	0	0	0
23	0	0	0	0	0	0	0	0	23	0	0	0	0	0	0	16	16
Total*	1	8	176	751	49	15	0	1000	Total*	3	664	152	46	101	18	16	1000

	GCS									GES							
	Included *s* hot-spot districts									Included *s* hot-spot districts							
l	6	7	8	9	10	11	12[#]	Total	*l*	6	7	8	9	10	11	12[#]	Total
11	0	0	0	0	0	16	0	16	11	0	0	0	0	1	10	0	11
12[#]	0	0	0	0	0	0	965	965	12[#]	0	0	0	0	0	60	0	60
13	0	0	0	0	0	1	2	3	13	0	0	0	0	0	86	283	369
14	0	0	0	0	0	0	14	14	14	0	0	0	0	0	20	243	263
15	0	0	0	0	0	0	0	0	15	0	0	0	0	0	4	234	238
16	0	0	0	0	0	0	1	1	16	0	0	0	0	0	0	53	53
17	0	0	0	0	0	0	0	0	17	0	0	0	0	0	0	5	5
18	0	0	0	0	0	0	0	0	18	0	0	0	0	0	0	1	1
Total*	0	0	0	0	0	17	983	1000	Total*	0	0	0	0	1	180	819	1000

CS: Circular spatial scan statistic, ES: Elliptic spatial scan statistic, GCS: Circular spatial scan statistic using Gini coefficient, GES: Elliptic spatial scan statistic using Gini coefficient, OF: Flexible spatial scan statistic, RC: Circular spatial scan statistic with a restricted likelihood ratio. RF: Flexible spatial scan statistic with a restricted likelihood ratio. 1000 trials were carried out. *The usual power is 1000/1000. [#]The number of districts in the true cluster for model B is 12.

Table C2. Estimated bivariate power distributions P(l,s) × 1,000 of the 7 methods for cluster model B (RR = 1.5) (continued).

	OF							
	Included *s* hot-spot districts							
l	6	7	8	9	10	11	12[#]	Total
11	0	0	0	0	0	0		
12[#]	0	0	0	0	0	0	998	998
13	0	0	0	0	0	0	2	2
Total*	0	0	0	0	0	0	1000	1000

	RC							
	Included *s* hot-spot districts							
l	6	7	8	9	10	11	12[#]	Total
11	0	0	0	0	0	8	0	8
12[#]	0	0	0	0	0	0	989	989
13	0	0	0	0	0	0	3	3
Total*	0	0	0	0	0	8	992	1000

	RF							
	Included *s* hot-spot districts							
l	6	7	8	9	10	11	12[#]	Total
12[#]	0	0	0	0	0	0	998	998
13	0	0	0	0	0	0	2	2
Total*	0	0	0	0	0	0	1000	1000

CS: Circular spatial scan statistic, ES: Elliptic spatial scan statistic, GCS: Circular spatial scan statistic using Gini coefficient, GES: Elliptic spatial scan statistic using Gini coefficient, OF: Flexible spatial scan statistic, RC: Circular spatial scan statistic with a restricted likelihood ratio. RF: Flexible spatial scan statistic with a restricted likelihood ratio. 1000 trials were carried out. *The usual power is 1000/1000. [#]The number of districts in the true cluster for model B is 12.

Table C3. Estimated bivariate power distributions P(l,s) × 1,000 of the 7 methods for cluster model C (RR = 2).

	CS									ES							
	Included *s* hot-spot districts									Included *s* hot-spot districts							
l	6-8	9	10	11	12	13	14#	Total	*l*	8	9	10	11	12	13	14#	Total
6	1	0	0	0	0	0	0	1	6	0	0	0	0	0	0	0	0
7	10	0	0	0	0	0	0	10	7	0	0	0	0	0	0	0	0
8	70	0	0	0	0	0	0	70	8	0	0	0	0	0	0	0	0
9	87	1	0	0	0	0	0	88	9	0	26	0	0	0	0	0	26
10	64	4	0	0	0	0	0	68	10	0	0	0	0	0	0	0	0
11	35	3	0	0	0	0	0	38	11	0	0	12	4	0	0	0	16
12	11	3	26	0	0	0	0	40	12	0	0	0	36	213	0	0	249
13	2	5	207	12	0	0	0	226	13	0	0	0	4	307	0	0	311
14#	1	0	0	218	1	0	0	220	14#	0	0	0	1	396	0	0	397
15	0	0	67	93	1	0	0	161	15	0	0	0	0	0	0	0	0
16	0	0	0	21	8	3	0	32	16	0	0	0	0	0	0	0	0
17	0	0	0	0	3	0	0	3	17	0	0	0	0	1	0	0	1
18	0	0	0	0	0	43	0	43	18	0	0	0	0	0	0	0	0
Total*	281	16	300	344	13	46	0	1000	Total*	0	26	12	45	917	0	0	1000

	GCS									GES							
	Included *s* hot-spot districts									Included *s* hot-spot districts							
l	8	9	10	11	12	13	14#	Total	*l*	8	9	10	11	12	13	14#	Total
	0	0	0	0	0	0	0	0	11	0	0	0	2	0	0	0	2
12	0	0	0	0	5	0	0	5	12	0	0	0	0	170	0	0	170
13	0	0	0	0	0	85	0	85	13	0	0	0	0	49	11	0	60
14#	0	0	0	0	0	52	159	211	14#	0	0	0	0	20	243	2	265
15	0	0	0	0	0	8	441	449	15	0	0	0	0	0	164	25	189
16	0	0	0	0	0	1	212	213	16	0	0	0	0	2	89	45	136
17	0	0	0	0	0	0	17	17	17	0	0	0	0	2	19	56	77
18	0	0	0	0	0	0	4	4	18	0	0	0	0	0	10	19	29
19	0	0	0	0	0	0	13	13	19	0	0	0	0	0	8	55	63
20	0	0	0	0	0	0	3	3	20-21	0	0	0	0	1	3	5	9
Total*	0	0	0	0	5	146	849	1000	Total*	0	0	0	2	244	547	207	1000

CS: Circular spatial scan statistic, ES: Elliptic spatial scan statistic, GCS: Circular spatial scan statistic using Gini coefficient, GES: Elliptic spatial scan statistic using Gini coefficient, OF: Flexible spatial scan statistic, RC: Circular spatial scan statistic with a restricted likelihood ratio. RF: Flexible spatial scan statistic with a restricted likelihood ratio. 1000 trials were carried out. *The usual power is 1000/1000. #The number of districts in the true cluster for model C is 14.

Table C3. Estimated bivariate power distributions P(l,s) × 1,000 of the 7 methods for cluster model C (RR = 2) (continued).

	OF									RC							
	Included *s* hot-spot districts									Included *s* hot-spot districts							
l	8	9	10	11	12	13	14#	Total	*l*	8	9	10	11	12	13	14#	Total
12	0	0	0	0	2	0	0	2	12	0	0	0	0	5	0	0	5
13	0	0	0	0	0	45	0	45	13	0	0	0	0	0	133	0	133
14#	0	0	0	0	0	18	23	41	14#	0	0	0	0	0	5	829	834
15	0	0	0	0	0	1	900	901	15	0	0	0	0	0	0	27	27
16	0	0	0	0	0	0	11	11	16	0	0	0	0	0	0	1	1
Total*	0	0	0	0	2	64	934	1000		0	0	0	0	5	138	857	1000

	RF							
	Included *s* hot-spot districts							
l	8	9	10	11	12	13	14*	Total
12	0	0	0	0	2	0	0	2
13	0	0	0	0	0	58	0	58
14#	0	0	0	0	0	2	901	903
15	0	0	0	0	0	0	37	37
Total*	0	0	0	0	2	60	938	1000

CS: Circular spatial scan statistic, ES: Elliptic spatial scan statistic, GCS: Circular spatial scan statistic using Gini coefficient, GES: Elliptic spatial scan statistic using Gini coefficient, OF: Flexible spatial scan statistic, RC: Circular spatial scan statistic with a restricted likelihood ratio. RF: Flexible spatial scan statistic with a restricted likelihood ratio. 1000 trials were carried out. *The usual power is 1000/1000. #The number of districts in the true cluster for model C is 14.

Table C4. Estimated bivariate power distributions P(l,s) × 1,000 of the 7 methods for cluster model D (RR = 2).

	CS									ES							
	Included *s* hot-spot districts									Included *s* hot-spot districts							
l	1	2	3	4	5	6	7#	Total	*l*	1	2	3	4	5	6	7#	Total
2	0	0	0	0	0	0	0	0	3	0	0	8	0	0	0	0	8
3	0	0	0	0	0	0	0	0	4	0	0	1	937	0	0	0	938
4	0	0	0	0	0	0	0	0	5	0	0	0	0	0	0	0	0
5	0	0	0	16	0	0	0	16	6	0	0	0	22	2	0	0	24
6	0	0	0	0	0	0	0	0	7#	0	0	0	18	0	0	0	18
7#	0	0	0	0	0	0	0	0	8	0	0	0	0	0	0	0	0
8	0	0	0	0	101	0	0	101	9	0	0	0	0	4	1	0	5
9	0	0	0	0	0	4	0	4	10	0	0	0	0	2	0	0	2
10	0	0	0	0	0	879	0	879	11	0	0	0	0	0	0	0	0
11	0	0	0	0	0	0	0	0	12	0	0	0	0	0	1	0	1
12	0	0	0	0	0	0	0	0	13	0	0	0	0	0	3	0	3
13	0	0	0	0	0	0	0	0	14	0	0	0	0	0	0	0	0
14	0	0	0	0	0	0	0	0	15	0	0	0	0	0	0	0	0
15	0	0	0	0	0	0	0	0	16	0	0	0	0	0	0	1	1
Total*	0	0	0	16	101	883	0	1000	Total*	0	0	9	977	8	5	1	1000

	GCS									GES							
	Included *s* hot-spot districts									Included *s* hot-spot districts							
l	1	2	3	4	5	6	7#	Total	*l*	1	2	3	4	5	6	7#	Total
7#	0	0	0	0	0	2	894	896	7#	0	0	0	0	0	0	357	357
8	0	0	0	0	0	1	40	41	8	0	0	0	0	0	0	212	212
9	0	0	0	0	0	0	57	57	9	0	0	0	0	0	0	368	368
10	0	0	0	0	0	0	1	1	10	0	0	0	0	0	0	51	51
11	0	0	0	0	0	0	1	1	11	0	0	0	0	0	0	5	5
12	0	0	0	0	0	0	0	0	12	0	0	0	0	0	0	6	6
13	0	0	0	0	0	0	1	1	13	0	0	0	0	0	0	1	1
Total*	0	0	0	0	0	6	994	1000	Total*	0	0	0	0	0	0	1000	1000

CS: Circular spatial scan statistic, ES: Elliptic spatial scan statistic, GCS: Circular spatial scan statistic using Gini coefficient, GES: Elliptic spatial scan statistic using Gini coefficient, OF: Flexible spatial scan statistic, RC: Circular spatial scan statistic with a restricted likelihood ratio. RF: Flexible spatial scan statistic with a restricted likelihood ratio. 1000 trials were carried out. *The usual power is 1000/1000. #The number of districts in the true cluster for model D is 7.

Table C4. Estimated bivariate power distributions P(l,s) × 1,000 of the 7 methods for cluster model D (RR = 2) (continued).

	OF									RC							
	Included *s* hot-spot districts									Included *s* hot-spot districts							
l	1	2	3	4	5	6	7#	Total	*l*	1	2	3	4	5	6	7#	Total
6	0	0	0	0	0	31	0	31	6	0	0	0	0	0	4	0	4
7#	0	0	0	0	0	0	490	490	7#	0	0	0	0	0	0	971	971
8	0	0	0	0	0	0	479	479	8	0	0	0	0	0	0	25	25
Total*	0	0	0	0	0	31	969	1000	Total*	0	0	0	0	0	0	1000	1000

	RF							
	Included *s* hot-spot districts							
l	1	2	3	4	5	6	7#	Total
6	0	0	0	0	0	11	0	11
7#	0	0	0	0	0	0	960	960
8	0	0	0	0	0	0	29	29
Total*	0	0	0	0	0	11	989	1000

CS: Circular spatial scan statistic, ES: Elliptic spatial scan statistic, GCS: Circular spatial scan statistic using Gini coefficient, GES: Elliptic spatial scan statistic using Gini coefficient, OF: Flexible spatial scan statistic, RC: Circular spatial scan statistic with a restricted likelihood ratio. RF: Flexible spatial scan statistic with a restricted likelihood ratio. 1000 trials were carried out. *The usual power is 1000/1000. #The number of districts in the true cluster for model D is 7.

Table C5. Estimated bivariate power distributions P(l,s) × 1,000 of the 7 methods for cluster model E (RR = 2).

	CS									ES							
	Included *s* hot-spot districts									Included *s* hot-spot districts							
l	12	13	14	15	16	17	18#	Total	*l*	11-12	13	14	15	16	17	18#	Total
12	0	0	0	0	0	0	0	0	11	8	0	0	0	0	0	0	8
13	0	0	0	0	0	0	0	0	12	12	0	0	0	0	0	0	12
14	0	0	0	0	0	0	0	0	13	10	0	0	0	0	0	0	10
15	0	0	0	0	0	0	0	0	14	0	3	0	0	0	0	0	3
16	0	0	3	6	0	0	0	9	15	0	158	0	17	0	0	0	175
17	0	0	0	0	279	16	0	295	16	0	2	77	0	14	0	0	93
18#	0	0	0	4	4	1	0	9	17	0	0	419	0	0	0	0	419
19	0	0	0	0	4	402	10	416	18#	0	0	0	213	0	4	0	217
20	0	0	0	0	0	17	0	17	19	0	0	0	1	1	0	0	2
21	0	0	0	0	0	2	133	135	20	0	0	0	0	58	0	0	58
22	0	0	0	0	0	7	7	14	21	0	0	0	0	2	0	0	2
23	0	0	0	0	0	0	1	1	22	0	0	0	0	0	0	0	0
24	0	0	0	1	0	0	0	1	23	0	0	0	0	0	0	0	0
25	0	0	0	0	0	103	0	103	24	0	0	0	0	0	0	0	0
26	0	0	0	0	0	0	0	0	25	0	0	0	0	0	1	0	1
Total*	0	0	3	11	287	548	151	1000	Total*	30	163	496	231	75	5	0	1000

	GCS									GES							
	Included *s* hot-spot districts									Included *s* hot-spot districts							
l	12	13	14	15	16	17	18#	Total	*l*	12	13	14	15	16	17	18#	Total
15	0	0	0	0	0	0	0	0	15	0	0	0	14	0	0	0	14
16	0	0	0	0	0	0	0	0	16	0	0	0	10	215	0	0	225
17	0	0	0	0	0	18	0	18	17	0	0	0	0	92	183	0	275
18#	0	0	0	0	0	5	945	950	18#	0	0	0	0	24	215	23	262
19	0	0	0	0	0	1	1	2	19	0	0	0	0	3	114	27	144
20	0	0	0	0	0	0	27	27	20	0	0	0	0	2	51	6	59
21	0	0	0	0	0	0	3	3	21	0	0	0	0	0	4	15	19
22	0	0	0	0	0	0	0	0	22	0	0	0	0	0	1	1	2
Total*	0	0	0	0	0	24	976	1000	Total*	0	0	0	24	336	568	72	1000

CS: Circular spatial scan statistic, ES: Elliptic spatial scan statistic, GCS: Circular spatial scan statistic using Gini coefficient, GES: Elliptic spatial scan statistic using Gini coefficient, OF: Flexible spatial scan statistic, RC: Circular spatial scan statistic with a restricted likelihood ratio. RF: Flexible spatial scan statistic with a restricted likelihood ratio. 1000 trials were carried out. *The usual power is 1000/1000. #The number of districts in the true cluster for model E is 18.

Table C5. Estimated bivariate power distributions P(l,s) × 1,000 of the 7 methods for cluster model E (RR = 2) (continued).

	OF									RC							
	Included *s* hot-spot districts									Included *s* hot-spot districts							
l	12	13	14	15	16	17	18#	Total	*l*	12	13	14	15	16	17	18#	Total
17	0	0	0	0	0	0	0	0	17	0	0	0	0	0	16	0	16
18#	0	0	0	0	7	6	0	13	18#	0	0	0	0	0	0	982	982
19	0	0	0	0	0	695	112	807	19	0	0	0	0	0	0	2	2
20	0	0	0	0	0	7	173	180	20	0	0	0	0	0	0	0	0
Total*	0	0	0	0	7	708	285	1000	Total*	0	0	0	0	0	16	984	1000

	RF							
	Included *s* hot-spot districts							
l	12	13	14	15	16	17	18#	Total
17	0	0	0	0	0	40	0	40
18#	0	0	0	0	0	0	958	958
19	0	0	0	0	0	0	2	2
Total*	0	0	0	0	0	40	960	1000

CS: Circular spatial scan statistic, ES: Elliptic spatial scan statistic, GCS: Circular spatial scan statistic using Gini coefficient, GES: Elliptic spatial scan statistic using Gini coefficient, OF: Flexible spatial scan statistic, RC: Circular spatial scan statistic with a restricted likelihood ratio. RF: Flexible spatial scan statistic with a restricted likelihood ratio. 1000 trials were carried out. *The usual power is 1000/1000. #The number of districts in the true cluster for model E is 18.

Table C6. Estimated bivariate power distributions P(l,s) × 1,000 of the 7 methods for cluster model F (RR = 2).

	CS									ES							
	Included *s* hot-spot districts									Included *s* hot-spot districts							
l	2	3	4	5	6	7	8[#]	Total	*l*	2	3	4	5	6	7	8[#]	Total
5	0	0	0	0	0	0	0	0	5	0	0	0	7	0	0	0	7
6	0	0	0	0	0	0	0	0	6	0	0	0	0	23	0	0	23
7	0	0	0	0	46	0	0	46	7	0	0	0	0	239	21	0	260
8[#]	0	0	0	0	0	112	0	112	8[#]	0	0	0	0	0	569	51	620
9	0	0	0	0	0	198	0	198	9	0	0	0	0	0	9	8	17
10	0	0	0	0	18	20	463	501	10	0	0	0	0	0	19	0	19
11	0	0	0	0	0	63	64	127	11	0	0	0	0	0	0	49	49
12	0	0	0	0	0	0	5	5	12	0	0	0	0	0	1	2	3
13	0	0	0	0	2	0	1	3	13	0	0	0	0	0	0	1	1
14	0	0	0	0	0	5	0	5	14	0	0	0	0	0	0	1	1
15	0	0	0	0	0	0	3	3	15	0	0	0	0	0	0	0	0
Total*	0	0	0	0	66	398	536	1000	Total*	0	0	0	7	262	619	112	1000

	GCS									GES							
	Included *s* hot-spot districts									Included *s* hot-spot districts							
l	2	3	4	5	6	7	8[#]	Total	*l*	2	3	4	5	6	7	8[#]	Total
6	0	0	0	0	0	0	0	0	6	0	0	0	0	16	0	0	16
7	0	0	0	0	0	0	0	0	7	0	0	0	0	4	203	0	207
8[#]	0	0	0	0	0	0	798	798	8[#]	0	0	0	0	1	48	356	405
9	0	0	0	0	0	0	91	91	9	0	0	0	0	0	27	213	240
10	0	0	0	0	0	0	83	83	10	0	0	0	0	0	15	51	66
11	0	0	0	0	0	0	21	21	11	0	0	0	0	0	7	26	33
12	0	0	0	0	0	0	3	3	12	0	0	0	0	0	3	19	22
13	0	0	0	0	0	0	3	3	13	0	0	0	0	0	1	5	6
14	0	0	0	0	0	0	1	1	14	0	0	0	0	0	0	3	3
15	0	0	0	0	0	0	0	0	15	0	0	0	0	0	0	1	1
16	0	0	0	0	0	0	0	0	16	0	0	0	0	0	0	1	1
Total*	0	0	0	0	0	0	1000	1000	Total*	0	0	0	0	21	304	675	1000

CS: Circular spatial scan statistic, ES: Elliptic spatial scan statistic, GCS: Circular spatial scan statistic using Gini coefficient, GES: Elliptic spatial scan statistic using Gini coefficient, OF: Flexible spatial scan statistic, RC: Circular spatial scan statistic with a restricted likelihood ratio. RF: Flexible spatial scan statistic with a restricted likelihood ratio. 1000 trials were carried out. *The usual power is 1000/1000. [#]The number of districts in the true cluster for model F is 8.

Table C6. Estimated bivariate power distributions P(l,s) × 1,000 of the 7 methods for cluster model F (RR = 2) (continued).

	OF							
	Included *s* hot-spot districts							
l	2	3	4	5	6	7	8[#]	Total
6	0	0	0	0	0	0	0	0
7	0	0	0	0	0	0	0	0
8[#]	0	0	0	0	0	0	0	0
9	0	0	0	0	0	0	38	38
10	0	0	0	0	0	0	942	942
11	0	0	0	0	0	0	20	20
12	0	0	0	0	0	0	0	0
13	0	0	0	0	0	0	0	0
Total*	0	0	0	0	0	0	1000	1000

	RC							
	Included *s* hot-spot districts							
l	2	3	4	5	6	7	8[#]	Total
6	0	0	0	0	0	0	0	0
7	0	0	0	0	0	0	0	0
8[#]	0	0	0	0	0	0	965	965
9	0	0	0	0	0	0	35	35
10	0	0	0	0	0	0	0	0
11	0	0	0	0	0	0	0	0
12	0	0	0	0	0	0	0	0
13	0	0	0	0	0	0	0	0
Total*	0	0	0	0	23	252	725	1000

	RF							
	Included *s* hot-spot districts							
l	2	3	4	5	6	7	8[#]	Total
6	0	0	0	0	0	0	0	0
7	0	0	0	0	0	0	0	0
8[#]	0	0	0	0	0	0	993	993
9	0	0	0	0	0	0	7	7
10	0	0	0	0	0	0	0	0
11	0	0	0	0	0	0	0	0
12	0	0	0	0	0	0	0	0
Total*	0	0	0	0	0	0	1000	1000

CS: Circular spatial scan statistic, ES: Elliptic spatial scan statistic, GCS: Circular spatial scan statistic using Gini coefficient, GES: Elliptic spatial scan statistic using Gini coefficient, OF: Flexible spatial scan statistic, RC: Circular spatial scan statistic with a restricted likelihood ratio. RF: Flexible spatial scan statistic with a restricted likelihood ratio. 1000 trials were carried out. *The usual power is 1000/1000. [#]The number of districts in the true cluster for model F is 8.

Table C7. Estimated bivariate power distributions P(l,s) × 1,000 of the 7 methods for cluster model G (RR = 2).

	CS									ES							
	Included *s* hot-spot districts									Included *s* hot-spot districts							
l	2	3	4	5	6	7	8#	Total	*l*	2	3	4	5	6	7	8#	Total
6	0	0	0	0	64	0	0	64	6	0	0	0	0	0	0	0	0
7	0	0	0	0	0	531	0	531	7	0	0	0	0	0	1	0	1
8#	0	0	0	0	0	0	0	0	8#	0	0	0	0	0	0	999	999
9	0	0	0	0	0	0	405	405	9	0	0	0	0	0	0	0	0
Total*	0	0	0	0	64	531	405	1000	Total*	0	0	0	0	0	1	999	1000

	GCS									GES							
	Included *s* hot-spot districts									Included *s* hot-spot districts							
l	2	3	4	5	6	7	8#	Total	*l*	2	3	4	5	6	7	8#	Total
7	0	0	0	0	0	7	0	7	7	0	0	0	0	0	0	0	0
8#	0	0	0	0	0	3	4	7	8#	0	0	0	0	0	0	696	696
9	0	0	0	0	0	2	781	783	9	0	0	0	0	0	1	43	44
10	0	0	0	0	0	0	138	138	10	0	0	0	0	0	0	168	168
11	0	0	0	0	0	0	28	28	11	0	0	0	0	0	0	27	27
12	0	0	0	0	0	1	32	33	12	0	0	0	0	0	0	44	44
13	0	0	0	0	0	0	4	4	13	0	0	0	0	0	0	5	5
14	0	0	0	0	0	0	0	0	14	0	0	0	0	0	0	11	11
15	0	0	0	0	0	0	0	0	15	0	0	0	0	0	0	3	3
16	0	0	0	0	0	0	0	0	16	0	0	0	0	0	0	2	2
Total*	0	0	0	0	0	13	987	1000	Total*	0	0	0	0	0	1	999	1000

CS: Circular spatial scan statistic, ES: Elliptic spatial scan statistic, GCS: Circular spatial scan statistic using Gini coefficient, GES: Elliptic spatial scan statistic using Gini coefficient, OF: Flexible spatial scan statistic, RC: Circular spatial scan statistic with a restricted likelihood ratio. RF: Flexible spatial scan statistic with a restricted likelihood ratio. 1000 trials were carried out. *The usual power is 1000/1000. #The number of districts in the true cluster for model F is 8.

Table C7. Estimated bivariate power distributions P(l,s) × 1,000 of the 7 methods for cluster model G (RR = 2) (continued).

	OF									RC							
	Included *s* hot-spot districts									Included *s* hot-spot districts							
l	2	3	4	5	6	7	8#	Total	*l*	2	3	4	5	6	7	8#	Total
7	0	0	0	0	0	2	0	2	7	0	0	0	0	0	4	0	4
8#	0	0	0	0	0	0	998	998	8#	0	0	0	0	0	0	993	993
9	0	0	0	0	0	0	0	0	9	0	0	0	0	0	0	3	3
Total*	0	0	0	0	0	2	998	1000	Total*	0	0	0	0	0	4	996	1000

	RF							
	Included *s* hot-spot districts							
l	2	3	4	5	6	7	8#	Total
7	0	0	0	0	0	2	0	2
8#	0	0	0	0	0	0	998	998
Total*	0	0	0	0	0	2	998	1000

CS: Circular spatial scan statistic, ES: Elliptic spatial scan statistic, GCS: Circular spatial scan statistic using Gini coefficient, GES: Elliptic spatial scan statistic using Gini coefficient, OF: Flexible spatial scan statistic, RC: Circular spatial scan statistic with a restricted likelihood ratio. RF: Flexible spatial scan statistic with a restricted likelihood ratio. 1000 trials were carried out. *The usual power is 1000/1000. #The number of districts in the true cluster for model G is 8.
